# Study of Degradation Kinetics and Structural Analysis of Related Substances of Ceftobiprole by HPLC with UV and MS/MS Detection

**DOI:** 10.3390/ijms232315252

**Published:** 2022-12-03

**Authors:** Dariusz Boczar, Katarzyna Bus, Katarzyna Michalska

**Affiliations:** 1 Department of Synthetic Drugs, National Medicines Institute, Chełmska 30/34, 00-725 Warsaw, Poland; 2 Department of Spectrometric Methods, National Medicines Institute, Chełmska 30/34, 00-725 Warsaw, Poland

**Keywords:** ceftobiprole, degradation, kinetic studies, stress-testing, LC/MS/MS

## Abstract

Ceftobiprole is a novel β-lactam antibiotic, active against methicillin-resistant *Staphylococcus aureus*, vancomycin-resistant *S. aureus* and penicillin-resistant *Streptococcus pneumoniae*. To artificially generate potential degradation products (DPs) of ceftobiprole that may be formed under relevant storage conditions, acidic, alkaline, oxidative, photolytic and thermolytic stress tests were performed in both solution and solid state. A novel selective HPLC method was developed for the separation of ceftobiprole from its DPs and synthesis by-products (SBPs) using Kinetex Biphenyl column, ammonium acetate buffer pH 5.8 and acetonitrile. The kinetic studies demonstrated the low stability of ceftobiprole in alkaline solution, in the presence of an oxidising agent and under irradiation with near UV. In the solid state, ceftobiprole underwent oxidation when the powder was irradiated with visible light and UV. Based on mass spectroscopic analysis, 13 new structural formulas of SBPs and DPs were proposed, along with molecular formulas for three other DPs obtained in solution and four oxidative DPs characteristic of solid-state degradation.

## 1. Introduction

**Figure 1:**
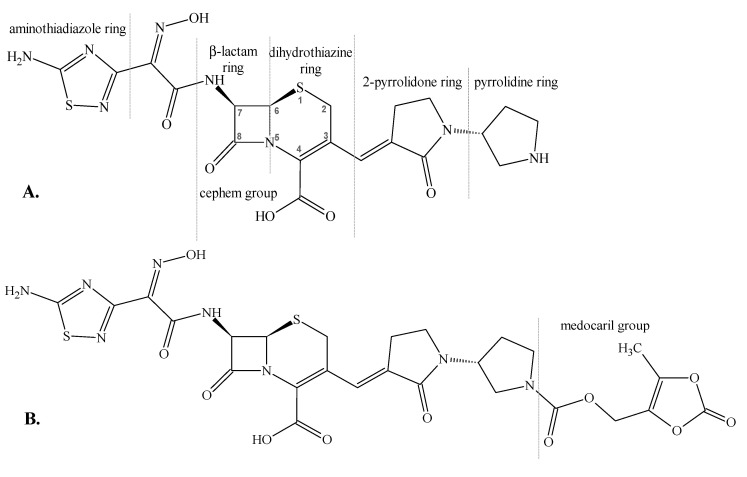
Molecular structures of ceftobiprole (**A**) and ceftobiprole medocaril (**B**).

Due to the continuous increase in bacterial resistance to antibiotics, there is an ongoing need to develop new effective drugs with antimicrobial activity. Historically, the first group of antibiotics, and the most numerous, are the β-lactams, which contain a β-lactam ring in their molecular structure and act by inhibiting the biosynthesis of the bacterial cell wall. Cephalosporins constitute the major subgroup of semi-synthetic β-lactams derived from 7-aminocephalosporanic acid and can be classified into five generations according to their activity against Gram-positive and Gram-negative bacteria [1]. Ceftobiprole (C_20_H_22_N_8_O_6_S_2_, Figure 1A) is a representative of the newest, fifth-generation antibiotics, next to ceftaroline [2] and ceftolozane [3,4]. It was approved and authorised in some countries on 20 October 2018 as the prodrug ceftobiprole medocaril (C_26_H_26_N_8_O_11_S_2_, Figure 1B) under the trade name of Zeftera© (previously Zevtera) or Mabelio©. Ceftobiprole had a rugged development and approval history, as Basilea Pharmaceutica’s partner failed to achieve United States Food and Drug Administration (FDA) and European Medicines Agency (EMA) approval. Therefore, Basilea Pharmaceutica bought back the rights and started new trials, finally succeeding in obtaining regulatory approval in many European Union countries and in Canada. More recently, on 28 June 2022, 13 years after the rejection of the first ceftobiprole registration attempt, Basilea Pharmaceutica were able to introduce ceftobiprole to the US market, due to a new Phase III clinical trial, the ERADICATE study, in adults with bacterial bloodstream infections caused by *Staphylococcus aureus*, where ceftobiprole was compared to daptomycin given with or without aztreonam (ClinicalTrials.gov identifier NCT03138733) [5,6]. Ceftobiprole is also under consideration for FDA approval for the treatment of acute bacterial skin and soft-tissue infections based on the results of the earlier TARGET study, which showed non-inferiority of the drug compared to vancomycin plus aztreonam (ClinicalTrials.gov identifier NCT03137173) [7].

Ceftobiprole was the first β-lactam to show in vitro activity against methicillin-resistant *S. aureus* (MRSA), vancomycin-resistant *S. aureus* (VRSA) and penicillin-resistant *Streptococcus pneumoniae* (PRP) [8]. The activity against Gram-negative bacteria corresponds to the known activity of other third-generation cephalosporins, such as ceftazidime. Hence, ceftobiprole is a new alternative to the empirical treatment of community-acquired pneumonia and non-ventilator-associated hospital-acquired pneumonia [9,10] when MRSA is proven or strongly suspected. Its broad-spectrum activity results from its high affinity to penicillin-binding proteins of Gram-positive bacteria, owing to the presence of the vinylpyrrolidone moiety, and its resistance to many different β-lactamases of Gram-negative pathogens due to the oxyimino aminothiazolyl substituent in the cephalosporin nucleus [11,12].

Stress testing of the active pharmaceutical ingredient (API) includes exposure to acidic, alkaline and oxidative conditions, as well as to photolysis and thermolysis, to artificially generate potential degradation products (DPs) that may or may not be formed under relevant storage conditions. Such a study is performed to elucidate the DP structures, establish degradation pathways and evaluate the intrinsic stability of the molecule. To avoid overstressing the ceftobiprole molecule, which may lead to degradation profiles that are not representative of real storage conditions, the proposed stress conditions were realistic and not excessive. The recommended duration of the stress-testing studies was performed to obtain 5–20% degradation. The stability-indicating assay method was carried out in accordance with the International Conference on Harmonisation (ICH) Guideline Q1A (R2) *Stability Testing of New Drug Substances and Products* [13] and recommendations given by Baertschi et al. in Section 2 of *Pharmaceutical Stress Testing* [14].

Ceftobiprole is a recent introduction to medicine; hence, there is limited literature data on analytical methods to test ceftobiprole and its DPs. A thin layer chromatographic-densitometric procedure was developed for the qualitative and quantitative analysis of ceftobiprole [15,16]. Stability studies of ceftobiprole have been carried out in solutions with different pH (1–13), showing that the degradation rate increased with increasing pH: the slowest in 0.1 M HCl (9.21 × 10^−3^ h^−1^ at 40 °C) and the fastest in 0.1 M NaOH (0.69 h^−1^ at 60 °C). However, due to technical limitations of the method, the molecular structure of the DPs was not elucidated [15]. Another approach used high performance liquid chromatographic (HPLC) methods with diode-array detection (DAD) [17] coupled with a mass spectrometer (MS) [18] to assay ceftobiprole in human plasma for routine therapeutic drug monitoring and pharmacokinetic studies. However, to the best of our knowledge, a study of degradation kinetics followed by the structural analysis of ceftobiprole DPs has not been published thus far.

The aim of this work consisted of four stages, namely: (i) to develop a new, selective HPLC/UV method that allows for the separation of ceftobiprole from its DPs based on stress-testing studies carried out in both solution and solid state, (ii) to use the developed method to study the intrinsic stability of ceftobiprole by calculating the degradation kinetics and determining the rate constants and activation energies ($E_{a}$), (iii) to propose the structures of DPs and synthesis by-products (SBPs) based on HPLC coupled to tandem mass spectrometry (LC/MS/MS), and (iv) to establish fragmentation pathways for these DP structures. Knowledge of the structure of the DPs combined with knowledge of the structure–activity relationship (SAR), which is an approach to find the relationship between the chemical structure (or structural properties) and the biological activity of the molecules under study, makes it possible to predict the antimicrobial activity (presence or absence) of the resulting DPs. SAR results for cephalosporins were provided by Sahoo and Banik, pointing out that the *cis*-configuration at position C-6 and C-7, the fusion of the β-lactam ring with the dihydrothiazine ring, and the olefinic linkage at position C-3 and C-4 are essential for antibacterial activity [19].

One of the challenges of this study was the low water solubility of ceftobiprole (experimentally determined as *approx.* 0.04 mg/mL), which is insufficient to acquire high-quality fragmentation mass spectra. In order to increase the solubility, it was necessary to use a water-miscible inert cosolvent; however, the effect of the inert cosolvent on hydrolysis had to be determined. According to Baertschi et al. [14], a water-miscible organic cosolvent such as dimethyl sulphoxide (DMSO) can be added to achieve the recommended concentration of 0.1–1 mg/mL. DMSO is considered to be a good general solvent, usually inert to drugs, completely miscible with water and useful under both acidic and basic conditions [14]. The main disadvantage of using DMSO is the absorption in the low UV region, which can be visible in the chromatogram as a UV peak [14]; however, our research was recorded in the near (long-wave) UV region. On the other hand, the presence of a cosolvent may modify the rate of hydrolysis or the degradation pathway, and this should be taken into account and verified. Another approach to improve the solubility of ceftobiprole relies on dissolving the drug in an acidic solution. The additional advantage of an acidic environment, as demonstrated by Binert-Kusztal et al. [15], is the enhanced stability of ceftobiprole towards hydrolysis, compared to neutral and alkaline aqueous solutions. Consequently, our paper compares two different solvents, namely 10% DMSO in water and 0.1 M HCl, in terms of the stability and possible degradation pathways of ceftobiprole under various stress conditions.

## 2. Results and Discussion

### 2.1. Method Optimisation

Ceftobiprole is a molecule with many ionisable function groups, which means that it has many pK_a_ values [20]. Since its DPs are unknown, it is difficult to theoretically predict the optimal pH of the mobile phase. For this reason, a systematic series of measurements were performed for each column, using a mobile phase pH range of 2.8–7.8 with one pH unit increments. Chromatograms of the ceftobiprole solutions and its DPs obtained by hydrolysis, photolysis, thermolysis and oxidation were recorded at each mobile phase pH and the chromatograms were then superimposed and compared.

**Figure 2:**
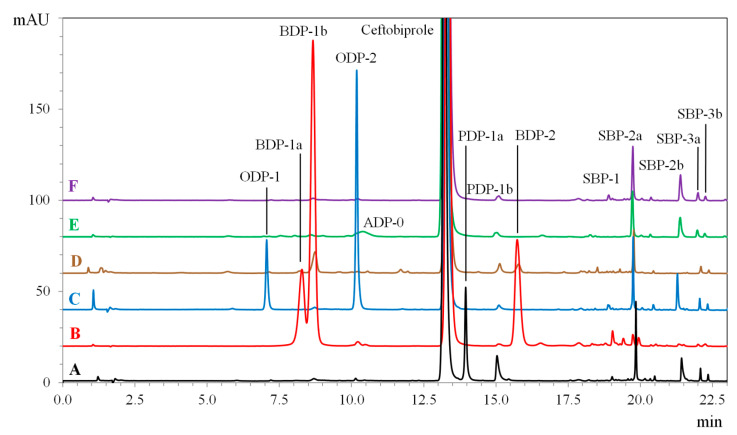
Overlaid chromatograms obtained by the separation of degradation products (DPs) of a 1 mg/mL ceftobiprole solution formed in different stress-testing studies: (**A**) photolysis for 6 h at 254 nm in 10% DMSO; (**B**) alkaline hydrolysis in 0.01 M NaOH for 45 min; (**C**) oxidation in 3% H_2_O_2_ in 10% DMSO for 90 min; (**D**) thermolysis at 70 °C for 270 min in 10% DMSO; (**E**) acidic hydrolysis in 0.1 M HCl for 6 h; (**F**) non-degraded solution of ceftobiprole in 10% DMSO. The first letter of the acronyms describing the DPs comes from the degradation conditions under which they were obtained (A—acidic, B—basic, O—oxidative, P—photolytic), and the acronym SBP denotes synthesis by-products.

Method development began with the use of a C18 column (Kinetex C18, 150 × 3 mm, 2.6 μm) and 5% ACN as the starting composition of the mobile phase. The separation of ceftobiprole from its DPs was unsuccessful, as the retention time of ceftobiprole was too short. In order to delay the elution of the main peak and thus enable the separation of DPs that are more polar than the parent ceftobiprole, columns compatible with 100% aqueous mobile phases were required. The optimal mobile phase pH and gradient program were chosen using Accucore AQ C18 (150 × 4.6 mm, 2.6 μm) and Kinetex PS C18 (150 × 2.1 mm, 2.6 μm) columns. However, the products of alkaline degradation could not be separated at any of the tested mobile phase pH values, either by changing the gradient run program (decreasing the gradient slope or replacing with an isocratic stage), increasing the column temperature or replacing ACN with MeOH. The applied modifications did not reduce the peak fronting and did not improve the resolution. Only the use of the Kinetex Biphenyl column made it possible to significantly improve the symmetry of the above-mentioned peaks. This column was previously used by Lima et al. [17] for the determination of ceftobiprole in human plasma but without performing any forced-degradation studies. The use of an elevated temperature (40 °C) was found to be crucial to obtain symmetric peak shapes of alkaline DPs. The experiments with different pH values for mobile phase A led to the conclusion that pH 5.8 was optimal for the separation of ceftobiprole and its related substances. Figure 2 presents the overlaid chromatograms of solutions of ceftobiprole and its DPs obtained by alkaline and acidic hydrolysis, photolysis, thermolysis and oxidation, where the DPs were separated using the optimal chromatographic conditions shown in Table 1.

The solubility of ceftobiprole in water has been experimentally found to be about 0.04 mg/mL, which is sufficient to analyse ceftobiprole and its DPs using a UV detector and an injection volume of 20 µL. However, it was impossible to record high-quality mass spectra of its DPs at such low concentration levels. Moreover, neither MeOH nor ACN (two solvents often used in HPLC analyses) provided greater solubility. Therefore, DMSO was selected from the list of recommended cosolvents proposed by Baertschi et al. [14], and a 10% aqueous solution was used as a solvent to enable a concentration of 1 mg/mL ceftobiprole to be obtained to make the LC/MS/MS study possible. Since the role of an organic cosolvent in the degradation under different conditions was initially unknown, the degradation of ceftobiprole was also performed in 0.1 M HCl, enabling the best stability of ceftobiprole in the pH range 1–13, according to Binert-Kusztal et al. [15]. The stability of ceftobiprole was therefore assessed in two solvents (10% DMSO and 0.1 M HCl), and the results obtained for ceftobiprole solutions stored at 25 °C for 3, 6, 12 and 24 h were compared (see Table 2). The percentage of drug remaining at time *t* was calculated by dividing the ceftobiprole peak area at time *t* by the ceftobiprole peak area at time 0. After 24 h, the decrease in ceftobiprole concentration was 1.1% in 10% DMSO and 10.4% in 0.1 M HCl, indicating that ceftobiprole is approximately ten times more stable in 10% DMSO.

The specificity of the method was confirmed in two different ways: the peaks of ceftobiprole and its unknown DPs were well separated from one another (Figure 2), and single peaks in the UV chromatogram corresponded to single components in the mass spectrum. The developed method allows for the determination of related substances at the level of 0.1 µg/mL (0.01% of 1 mg/mL) with a signal-to-noise ratio (S/N) of about 18. The linearity of the method was confirmed in the investigated range from 0.1 µg/mL to 1.2 mg/mL, with R^2^ = 0.9992.

### 2.2. Kinetics of Ceftobiprole Degradation in Solution-State Studies

In the series of kinetic experiments, the concentrations of ceftobiprole and its DPs under different stress conditions were monitored as a function of time. For the degradation of ceftobiprole, pseudo-first-order kinetics was assumed and the rate constant k was determined as the slope of the line fitted according to the following equation:

(1) $$\ln\left(\frac{a}{a_{0}}\right)=-kt$$

where $a_{0}$ is the initial peak area of ceftobiprole, and a is the peak area of ceftobiprole at time t. This method of calculation relies on the linearity and instrumental precision of the method, both of which were proven throughout the study, and does not require any additional assumptions.

In specific cases, such as when the peak area at *t* = 0 min is difficult to measure (e.g., immediately after dissolution or the addition of the reagent), lna was plotted against t and a straight line was fitted with two fitting parameters ($\ln a_{0}$ and k), as follows:

(2) $$\ln a=\ln a_{0}-kt$$

The concentration of DPs formed in solution at time *t* was calculated as a quotient of the peak area of the corresponding DP at time *t* and the initial peak area of ceftobiprole at time 0. This method of calculation relies on the assumption that ceftobiprole and its DPs have equal (or very similar) response factors. In other words, the peak areas of substances of the same concentration should be equal, regardless of whether it is ceftobiprole or its DP.

The shelf-life value *t*_90_, i.e., the time at which 90% of the initial drug concentration remains, was derived by substituting a/a_0_ = 0.9 in Equation (1), yielding:

(3) $$t_{90}=-\frac{\ln0.9}{k}$$

For the thermolytic reactions carried out at three different temperatures (50, 60 and 70 °C), the activation energy $E_{a}$ was estimated by means of the Arrhenius equation:

(4) $$\ln k=\;\frac{-E_{a}}{RT}+\ln A$$

where R is the universal gas constant 8.314 J/(mol × K), T(K) is the temperature and A is the pre-exponential factor, depending on how often molecules collide with one another.

Table 3 summarises the experimental stress conditions in the solution-state degradation studies, the percentage of DPs formed after 6 h of degradation and the calculated degradation rate constants. The coefficient of determination (R^2^) was generally not lower than 0.98, which proves that the kinetics of the tested reactions are consistent with the pseudo-first-order model. The first letter of the proposed acronyms of the DPs originates from the degradation conditions in which they are obtained (A—acidic, B—basic, O—oxidative, P—photolytic, T—thermolytic). The reactions proceeded long enough to find the optimum conditions, in which the highest amounts of DPs are formed, and this knowledge was applied in the sample preparation for the MS study, to obtain the best possible quality for the mass spectra.

In the chromatogram of the non-degraded sample (Figure 2, line F), six peaks of related substances of ceftobiprole were detected. Further studies showed that one of these impurities could be formed artificially by irradiation with a UV lamp with a wavelength of 366 nm, and was therefore designated as photolytic DP (PDP), while the other could not be formed under any applied stress conditions. Mass spectrometric analysis showed that they can be considered as three SBPs, two of which occur as pairs of isomers. The content of the related substances in the non-degraded solution of ceftobiprole is as follows: PDP-1b (0.25%), SBP-1 (0.22%), two isomers of SBP-2: SBP-2a (1.31%) and SBP-2b (0.88%), and two isomers of SBP-3: SBP-3a (0.21%) and SBP-3b (0.11%).

#### 2.2.1. Acidic Degradation Products (ADPs)

The recommended environment for acidic degradation is 0.1 M HCl [14]. However, ceftobiprole undergoes relatively slow degradation in this solvent, with a decrease of about 3% after 6 h (see Table 2). As can be seen in Figure 2, acidic hydrolysis performed in 0.1 M HCl, even for 6 h, led to the formation of very small quantities of DPs (the main ADP, which has been denoted as ADP-0, reaches 0.94% after 6 h). Therefore, in order to artificially generate significant amounts of DPs by the reaction of ceftobiprole with HCl, the acid concentration was increased 10-fold to 1 M, and the samples were incubated in a water bath at elevated temperature (50, 60 and 70 °C).

**Figure 3:**
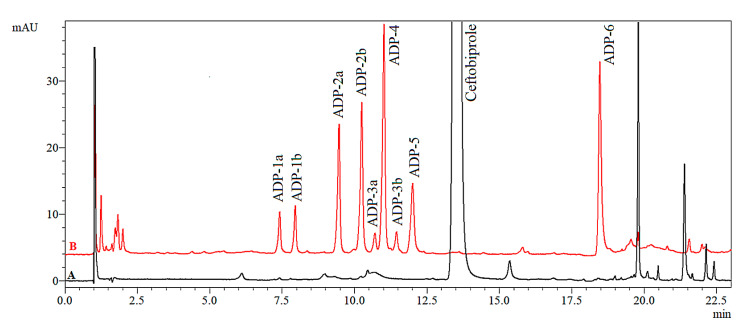
Overlaid chromatograms obtained from the separation of a 1 mg/mL solution of ceftobiprole by acidic hydrolysis in 1 M HCl: (**A**) at time 0; (**B**) after incubation at 70 °C for 360 min.

**Figure 4:**
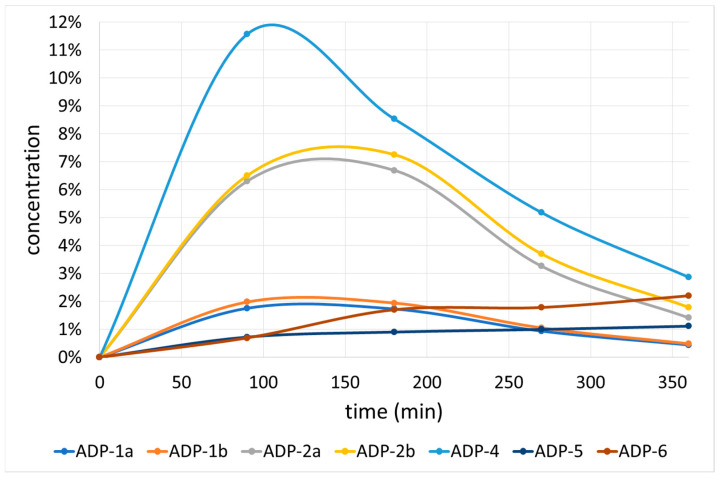
Time dependence of ceftobiprole decomposition in 1 M HCl at 70 °C.

Six DPs were formed in 1 M HCl: three of them (ADP-1, ADP-2 and ADP-3) eluted in two peaks of stereoisomers, whereas the remaining DPs, marked with the acronyms ADP-4, ADP-5 and ADP-6 were present as single peaks (Figure 3). ADP-0, obtained at room temperature, decomposed at elevated temperatures. At 50 °C, the isomers of ADP-1 and ADP-2 and the single peak of ADP-4 were formed during 6 h of reaction. Increasing the temperature to 60 °C resulted in the formation of further DPs, ADP-5 and ADP-6; however, their concentration was still very low after 6 h of heating. To obtain sufficient concentrations of these ADPs, the process temperature was increased by another 10 °C, to 70 °C. The time-dependence of the ADP concentrations in 1 M HCl at 70 °C is shown in Figure 4. ADPs 1–4 achieved maximum amounts after about 2 h of heating, whereas the content of ADPs 5 and 6 increased continuously.

The rate constants were determined for the degradation of ceftobiprole in 1 M HCl at 50, 60 and 70 °C, and the results are presented in Table 3. The Arrhenius plot was prepared using Equation (4) [21]. The $E_{a}$ can be considered as the magnitude of the energy barrier separating the minima of the potential energy surface corresponding to the initial and final thermodynamic state. For a chemical reaction to proceed at a reasonable rate, the temperature of the system should be high enough to enable the existence of an appreciable number of molecules with translational energy equal to or greater than the $E_{a}$. From a different point of view, the Arrhenius $E_{a}$ indicates the sensitivity of the reaction rate to temperature. The Arrhenius equation (Equation (4)) allows rate constants to be predicted at different temperatures, e.g., to extrapolate the linear plot to calculate the rate constant at room temperature [14]. The determined $E_{a}$ is equal to 102.3 kJ/mol (24.5 kcal/mol). The extrapolation of the obtained Arrhenius plot to 25 °C yielded a rate constant of 1.77 × 10^−6^ s^−1^, which corresponds to a shelf-life *t*_90_ value of 16.6 h in 1 M HCl at 25 °C.

#### 2.2.2. Basic Degradation Products (BDPs)

Ceftobiprole is extremely unstable in an alkaline environment. When ceftobiprole was dissolved in 0.1 M NaOH, the main peak disappeared completely in less than 45 min. Therefore, measuring the kinetics of the reaction at intervals of several minutes would be affected by a large error. In order to perform the kinetic studies accurately, the reaction was slowed down by reducing the concentration of 0.1 M NaOH to 0.01 M.

**Figure 5:**
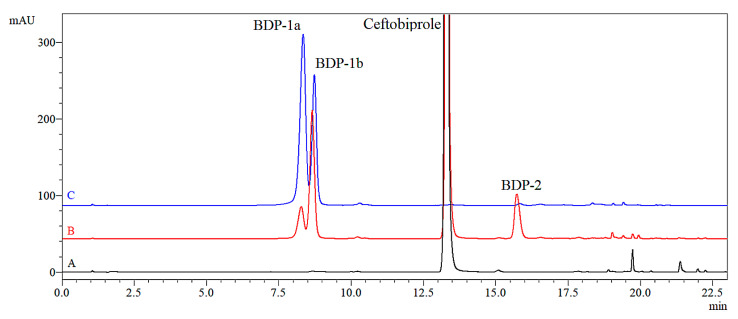
Overlaid chromatograms obtained from the separation of a 1 mg/mL ceftobiprole solution by alkaline hydrolysis in 0.01 M NaOH: (**A**) at time 0; (**B**) after 45 min; (**C**) after 720 min.

**Figure 6:**
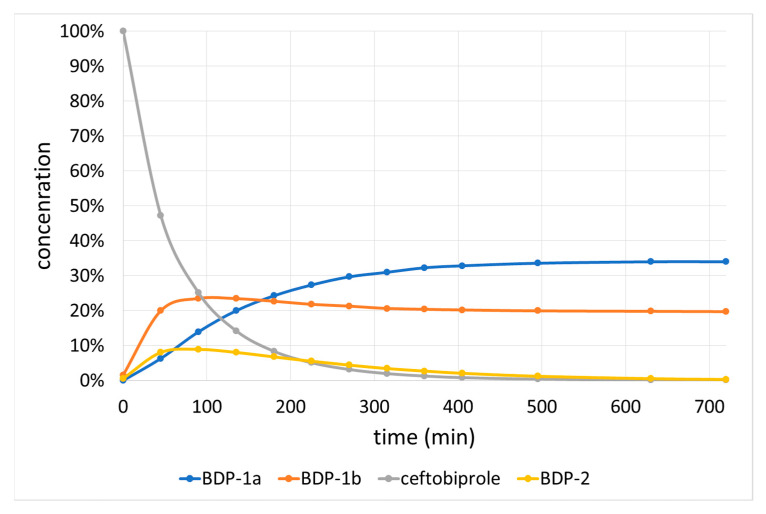
Time dependence of ceftobiprole decomposition in 0.01 M NaOH.

Two DPs were formed in 0.01 M NaOH: one pair of stereoisomers of open-ring ceftobiprole (BDP-1a, BDP-1b) and a lacton (BDP-2). The superimposed chromatograms for the solution analysed at specified time intervals are shown in Figure 5, while the time dependence of the BDP concentrations is presented in Figure 6. BDP-1b and BDP-2 reached their maximum levels after approximately 90 min, before slowly decreasing. The content of BDP-1a increased steadily during the measurement time. The degradation rate constant obtained in 0.01 M NaOH is the highest among the reactions carried out at 25 °C. Due to the difficulties in determining t = 0 min, Equation (2) was used in place of Equation (1).

#### 2.2.3. Oxidative Degradation Products (ODPs)

**Figure 7:**
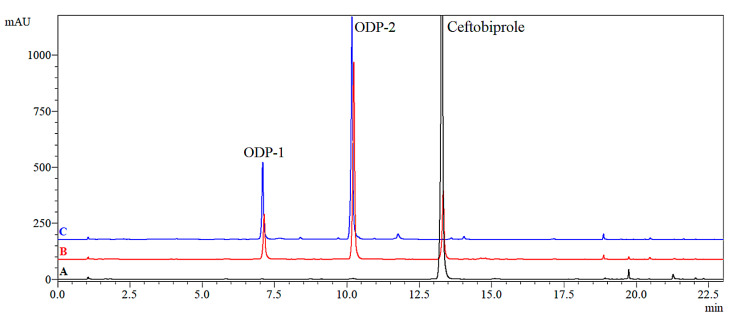
Overlaid chromatograms obtained from the separation of a 1 mg/mL ceftobiprole solution by oxidation with 3% H_2_O_2_: (**A**) ceftobiprole in 10% DMSO at time 0; (**B**) in 10% DMSO after 1080 min (18 h); (**C**) in 0.1 M HCl after 1080 min.

**Figure 8:**
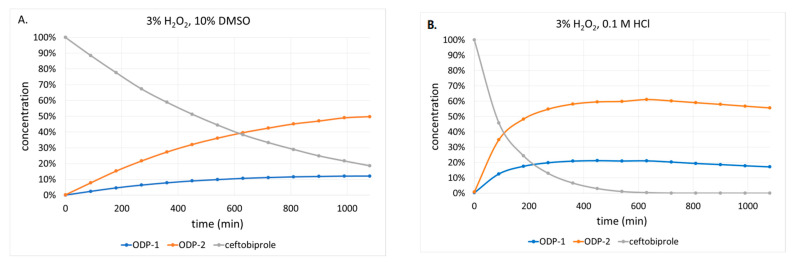
Time dependence of ceftobiprole decomposition in 3% H2O2 in: (**A**) 10% DMSO; (**B**) 0.1 M HCl.

Oxidation of an API can be caused by exposure to peroxides, which can be found in varying amounts in certain excipients, such as polysorbates, polyethylene glycol, povidone and hydroxypropyl cellulose [14]. Thus, it is common for drug substances to be exposed to peroxides after formulation and during storage and distribution. The oxidative degradation of ceftobiprole was carried out with 3% H_2_O_2_ in two different solvents. It appears that using 10% DMSO instead of 0.1 M HCl as a solvent to increase the solubility of the API may be preferable, as ceftobiprole is more stable in this solvent; however, there is a possibility that DMSO may be oxidised to methanesulphinic and methanesulphonic acids by ^·^OH radicals [22]. Thus, the reaction was performed in two solvents, and their kinetics were compared (Table 3). The reaction was five times faster in 0.1 M HCl than in 10% DMSO. There are two possible reasons for this: (i) the reaction rate slowing down in 10% DMSO due to the competitive oxidation of DMSO to methanesulphinic and methanesulphonic acids, or (ii) an acceleration of the reaction in 0.1 M HCl due to the involvement of H^+^ cations in the oxidation mechanism. Therefore, an additional experiment was performed without the addition of a cosolvent, at neutral pH. The degradation kinetics of a 0.04 mg/mL solution of ceftobiprole in 3% H_2_O_2_ in water showed that the time-dependence of the ceftobiprole peak area can be approximated by the first-order model during the first 360 min of the process, with a rate constant of 7.07 × 10^−5^ s^−1^. This value lies between the values obtained in 10% DMSO (2.56 × 10^−5^ s^−1^) and 0.1 M HCl (1.33 × 10^−4^ s^−1^), demonstrating that both of the above-discussed mechanisms (the slowdown in 10% DMSO and the acceleration in 0.1 M HCl) affect the determined rate constants. The rate constant and $t_{90}$ of oxidation in 0.1 M HCl were of the same order of magnitude as for alkaline hydrolysis, indicating that ceftobiprole is particularly prone to the action of bases and oxidising agents. There were two main DPs, ODP-1 and ODP-2, which formed regardless of whether the process was carried out in 10% DMSO or 0.1 M HCl (Figure 7). In 10% DMSO, the concentration of the two main DPs continued to increase throughout the tested time (18 h), while their content reached a maximum after 7.5–10.5 h in 0.1 M HCl and then decreased (Figure 8).

#### 2.2.4. Photolytic Degradation Products (PDPs)

The UV–VIS spectrum of ceftobiprole has two maximums of absorption, at 230 and 320 nm. This indicates that the molecule absorbs radiation of near ultraviolet wavelengths and may be photolabile. In order to verify this hypothesis, solutions of ceftobiprole in 10% DMSO in water or 0.1 M HCl were irradiated at 254 and 366 nm.

**Figure 9:**
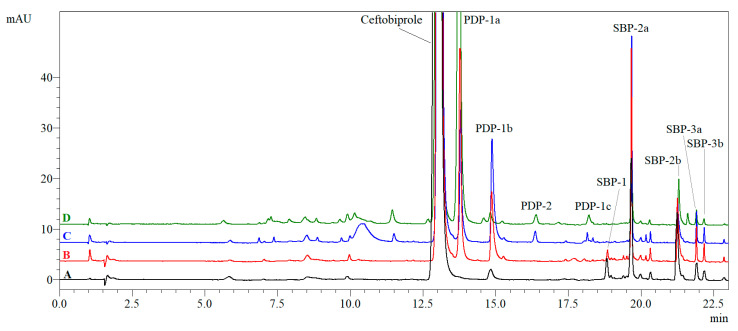
Overlaid chromatograms obtained from the separation of a 1 mg/mL ceftobiprole solution by photolytic degradation: (**A**) in 10% DMSO at time 0; (**B**) irradiation at λ = 254 nm in 10% DMSO after 360 min; (**C**) irradiation at λ = 254 nm in 0.1 M HCl after 360 min; (**D**) irradiation at λ = 366 nm in 0.1 M HCl after 360 min.

Figure 9 presents the overlaid chromatograms of ceftobiprole and its PDPs formed under different combinations of solvent and irradiation wavelength. The determined rate constants show that the degradation is comparable in terms of the solvents used, but about seven times faster at 366 than at 254 nm (Table 3). During photolytic process, three isomers of ceftobiprole (PDP-1a, b and c) were formed, along with another compound with one less sulphur atom compared to ceftobiprole (PDP-2). As can be seen in Figure 9 and Table 3, the same DPs were formed regardless of the solvent used or the wavelength of irradiation, but their relative amounts were different. Mainly PDP-1a was formed after 6 h irradiation at 366 nm, at a level of 15.48% in 10% DMSO and 10.61% in 0.1 M HCl. When using 254 nm, there was also a significant increase in PDP-1b, from 0.25% in the non-degraded sample to 1.26% in 10% DMSO and 1.90% in 0.1 M HCl after 6 h. The use of 0.1 M HCl as a solvent resulted in the formation of PDP-1c and PDP-2. SBPs are photolabile, as is ceftobiprole, and their peak areas decreased with time.

#### 2.2.5. Thermolytic Degradation

The Q1A (R2) guideline recommends testing the effect of temperature in 10 °C increments (50 °C, 60 °C, etc.) above a certain temperature in accelerated tests (40 °C) [13], while Baertschi et al. suggest avoiding temperatures above 70 °C due to the possibility of different decomposition pathways for some compounds [14]. Consequently, three temperatures, 50, 60 and 70 °C, were chosen, and the Arrhenius plot was prepared using these three points. The rate constants are summarised in Table 3. The determined $E_{a}$ is equal to 106.3 kJ/mol (25.4 kcal/mol, R² = 0.9987). The extrapolation of the obtained Arrhenius plot to 25 °C yielded a rate constant of 9.74 × 10^−8^ s^−1^, which corresponds to a shelf-life $t_{90}$ value of 12.5 days in 10% DMSO. A similar $E_{a}$ value was obtained under both thermolysis and acidic hydrolysis conditions (106.3 kJ/mol for $E_{a}$ in 10% DMSO and 102.3 kJ/mol in 1 M HCl), although the rate constant is about 15 times lower for 10% DMSO compared to 0.1 M HCl. The difference in the reaction rate should therefore be attributed to the difference in pre-exponential factor A in Equation (4), while a slight difference in $E_{a}$ (4 kJ/mol) has a significantly lower impact.

**Figure 10:**
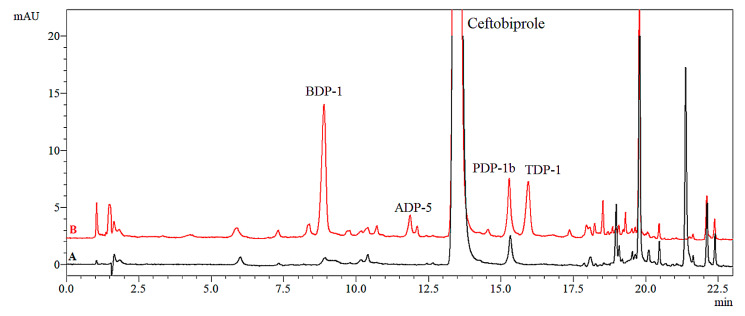
Overlaid chromatograms obtained from the separation of 1 mg/mL ceftobiprole solution by thermolysis in 10% DMSO: (**A**) at time 0; (**B**) after 270 min at 70 °C.

Four DPs were formed during thermolysis, three of which (ADP-5, BDP-1 and PDP-1b) were also produced by acidic hydrolysis, alkaline hydrolysis and photolysis, respectively. BDP-1 is a DP that forms predominantly (Figure 10). After 6 h of heating at 50 °C, its content reached 0.45%, whilst reaching 1.32% at 60 and 70 °C. At higher temperatures, the PDP-1b content also increased (after 6 h: 0.31% at 60 °C and 0.60% at 70 °C), and a new isomer of ceftobiprole formed, TDP-1, reaching 0.15% at 60 °C and 0.83% at 70 °C. Heating to 70 °C additionally led to the formation of ADP-5. After more than 6 h of thermolysis, a decrease in BDP-1, PDP-1b and TDP-1 content was observed, while mainly ADP-5 remained and increased over time.

Although the total content of the identified DPs appeared to be small (e.g., 3.10% after 6 h at 70 °C, as shown in Table 3), the percentage of ceftobiprole remaining, determined by dividing its peak area at time t by the peak area at time 0, drastically decreased: after 6 h it was 95.70% at 50 °C, 81.78% at 60 °C and 55.83% at 70 °C. This means that the content of ceftobiprole and its identified DPs does not add up to 100%. There are two possible explanations: (i) the effective molar absorption coefficient at 320 nm is different for the resulting DPs and ceftobiprole, and/or (ii) there are additional thermolytic DPs deprived of a chromophore and therefore undetectable at 320 nm. The recommendation to carry out the process long enough to obtain 5–20% degradation [14] is problematic in the case of thermolysis, as we have a slight increase in the content of impurities and, at the same time, a large decrease in the peak area for ceftobiprole. Although more than 20% of the ceftobiprole was degraded, the calculated sum of the identified DPs was still less than 5%.

### 2.3. Solid-State Degradation Kinetics

To determine the degradation rate constants in the solid-state experiments, the peak area of ceftobiprole in the degraded solution cannot be simply divided by the corresponding area in the reference, since the two solutions were prepared with different analytical weights. Therefore, the following procedure was applied: the area of ceftobiprole was divided by the sum of all peaks in the chromatogram, excluding peaks of SBPs; then, the natural logarithm of this ratio was plotted against time, and the rate constant k was determined using Equation (1). The concentration of DPs formed in the solid state at time *t* was calculated as a quotient of the peak area of the corresponding DP at time *t* and the sum of all peaks in the same chromatogram, excluding the peaks of SBPs.

The degradation rate constants in the solid state were calculated for various stress conditions, and the percentages of the formed DPs were determined assuming the equality of the response factors (Table 4). The rate constants ranged from 3.61 × 10^−9^ s^−1^ (50 °C, 75% RH) to 1.68 × 10^−7^ s^−1^ (photolytic, VIS) and are thus several orders of magnitude lower than those obtained for the degradation in solution (2.62 × 10^−6^ to 3.99 × 10^−4^ s^−1^). The solid-state experiments can be divided into two groups: thermolytic (an elevated temperature, with or without control of humidity), in which the formation of BDP-1b was observed, and photolytic (irradiation with a lamp producing visible light or UV at 366 nm), where several oxidative degradation products (ODPs) were generated.

Under thermolytic conditions, ceftobiprole undergoes degradation that leads to the formation of the following DPs: BDP-1b, PDP-1b and ODP-2. The first two DPs were also observed under thermolysis conditions carried out in solution, while ODP-2 is the main DP formed in the reaction of ceftobiprole with 3% H_2_O_2_ in solution. The latter was observed in solid-state thermolysis probably because the elevated temperature lowers the energetic barrier of the reaction between ceftobiprole powder and oxygen in the air. The increase in humidity lowers the degradation rate constant and promotes the formation of open-ring ceftobiprole (BDP-1b).

In the case of photolytic conditions, the DP profile is quite different. There was a measurable increase in the content of PDP-1b (which was also reported in photolytic experiments performed in solution) but also the formation of five isomers of ODPs in which one oxygen atom is added to the ceftobiprole molecule. Two of these isomers, ODP-1 and ODP-2, are products of the reaction of ceftobiprole with 3% H_2_O_2_, but another three, ODP-S1, ODP-S2 and ODP-S3, where the letter “S” denotes the solid state, did not form in significant amounts in solution. Finally, a new dimeric DP denoted as ODP-S4, not observed in previous experiments, formed under photolytic conditions with visible light and UV at 366 nm. There are several possible mechanisms for photoinduced oxidation, including conversion of the API to peroxy radicals, reaction of the API with excited-state singlet oxygen or reaction of the API radical cation with a superoxide anion [23].

The fastest photodegradation (rate constant of 1.68 × 10^−7^ s^−1^) was achieved with the use of a fluorescent lamp of 5260 lux. For confirmatory photostability studies, ICH guideline Q1B recommends that samples should be exposed to light providing an overall illumination of not less than 1.2 million lux hours [24]. This condition has been fulfilled, as 10 and 21 days of illumination with the lamp used in this study correspond to the exposure of 1.26 × 10^6^ and 2.65 × 10^6^ lux hours, respectively. The exposure of the solution of ceftobiprole to the same source of white light did not result in any measurable degradation of ceftobiprole. This demonstrates that the mechanism of photolysis in the solid state is completely different from that in solution, probably due to the relatively low concentration of accessible oxygen dissolved in water.

The rate constant for solid-state photolysis at 366 nm was 180 times slower than for solution-state photolysis. However, these values are difficult to compare as they relate to different reactions leading to different DPs. The irradiation of ceftobiprole solution at 366 nm led to the predominant formation of PDP-1a, which was not observed in significant amounts in the analogous solid-state experiment.

### 2.4. Mass Spectrometric Characterisation of the Impurities of Ceftobiprole

**Scheme 1:**
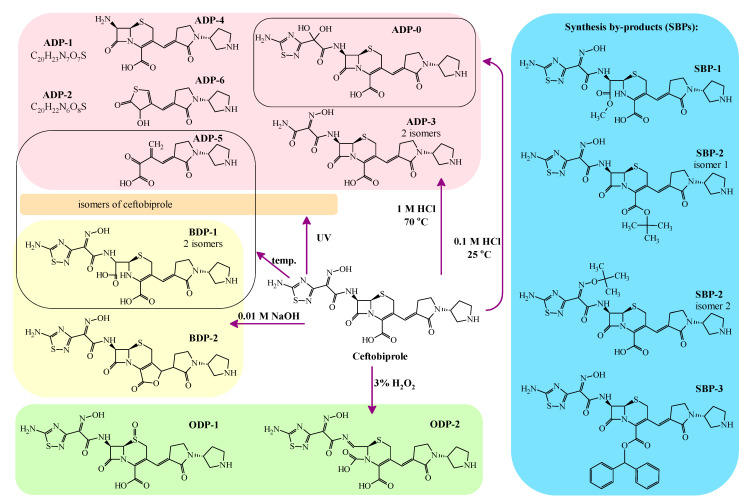
Structure of DPs of ceftobiprole obtained under different degradation conditions together with the synthesis by-products (SBPs).

Monoisotopic masses of the studied compounds were assigned based on the peaks [M + H]^+^, [M + K]^+^ (separated by about 37.9559 Da) and [M + 2H]^2+^. In the case of ceftobiprole, [3M + 2H]^2+^ (802.1716 Da) and [2M + H]^+^ (1069.2266 Da) adducts were also observed. The proposed structures of the identified related substances are presented in Table 5, while their fragmentation mass spectra and proposed fragmentation pathways are given in Figure S1 in Supplementary Materials. The fragmentation pathway for ceftobiprole is proposed in Figure S1A. The study was performed in positive ion mode; therefore, the *m/z* values given in the text and the table refer to the [M + H]^+^ species. In the proposed fragmentation pathways, structural and molecular formulas are generally given for neutral molecules M, not protonated ones, because it is difficult to predict where exactly a proton has been attached. In such cases the *m/z* of [M + H]^+^ was calculated to allow for comparison with the corresponding experimental *m/z* in the fragmentation mass spectrum. However, in some cases, it was easier to propose a carbocation structure instead, and then, the monoisotopic mass of the cation can be directly compared with the *m/z* in the spectrum. The names of the rings discussed below are shown in the structural formula of ceftobiprole in Figure 1A together with the numbering of atoms in the cephem ring. Relative retention times (RRTs) were calculated using data obtained with a Nexera HPLC/UV chromatographic system. Scheme 1 summarises the proposed structural formulas of DPs obtained under different degradation conditions, together with the SBPs of ceftobiprole.

#### 2.4.1. Identification of Synthesis By-Products (SBPs)

SBPs were identified with the aid of the patent review presenting the most probable synthesis pathway of ceftobiprole, patented by Basilea, the manufacturer of the ceftobiprole used in this study [25].

SBP-1, *m/z* 567.1431, can be described with a molecular formula [C_21_H_26_N_8_O_7_S_2_ + H]^+^, which means that SBP-1 possesses one carbon, one oxygen and four hydrogen atoms more than ceftobiprole. This observation can be explained by the assumed methanolysis, i.e., the hydrolysis of the β-lactam ring (which increases the molecular formula by H_2_O), followed by esterification with a methyl group (which further increases the molecular formula by CH_2_). According to the fragmentation mass spectrum (Figure S1B), the easy loss of H_2_S (also observed in the case of BDP-1, discussed below) can serve as evidence for the breakdown of the bicyclic cephem moiety, whereas the loss of only one CO_2_ suggests that there is only one free carboxylic group and that the second one is bound (e.g., esterified). The fragment ions of *m/z* 308 and below are the same as for ceftobiprole, which indicates that the right-hand part of the SBP-1 molecule (the dihydrothiazine, 2-pyrrolidone and pyrrolidine rings) is the same as for ceftobiprole. This indicates that the carboxyl group attached to the dihydrothiazine group is unbound, whereas the carboxyl group formed as a result of β-lactam hydrolysis is part of an ester moiety.

SBP-1 decomposes under all stress conditions, and no increase in the content of this related substance was observed during the stress aging tests. After dissolving ceftobiprole in water, this impurity was detectable, but after dissolving the sample in 0.1 M HCl, the SBP-1 peak was not observed. Given the fact that SBP-1 possesses an open β-lactam ring, which indicates the degradation process, but has not been artificially generated under any applied stress conditions, it can be assumed that SBP-1 is formed in a side (degradation) reaction occurring in the synthesis process, and therefore, it has been classified as a synthesis by-product, i.e., the product of a side reaction.

SBP-2, which eluted in two peaks of *m/z* 591.1791 (a) and 591.1799 (b), has the largest intensity in the chromatogram of the non-degraded sample. The proposed formula [C_24_H_30_N_8_O_6_S_2_ + H]^+^ differs from ceftobiprole by C_4_H_8_, which can be an isomer of the butyl group. In the synthetic route, a *tert*-butyloxycarbonyl group (Boc) is used as a protecting group, attached to the secondary amine in the pyrrolidine ring [25]. It has been reported elsewhere that *tert*-butyl cations may form as a side product during the process of deprotection with trifluoroacetic acid (TFA) and these cations may attack the nucleophilic sites of a molecule [26]. Hence, two structures were proposed, with a *tert*-butyl group attached to the carboxylic and oxime groups, respectively. Ceftobiprole and its fragmentation ions are the main signals in the fragmentation mass spectrum of SBP-2 (Figure S1C), and therefore, it was impossible to assign which peak corresponds to which isomer.

SBP-3 elutes in two peaks of *m/z* 701.1926 (a) and 701.1933 (b), which correspond to [C_33_H_32_N_8_O_6_S_2_ + H]^+^. The difference between the formulas of SBP-3 and ceftobiprole is C_13_H_10_, which also appears in the fragmentation mass spectrum as the strongest signal, located at *m/z* 167 (Figure S1D). It can be explained as a diphenylmethyl (C_6_H_5_)_2_CH_2_ group, which is used in the synthetic process as a protecting group attached to the carboxyl moiety. Therefore, SBP-3 remains as the impurity of the final product due to the incomplete removal of the above mentioned protecting group.

#### 2.4.2. Identification of ADPs

ADP-0 is the DP which forms in 0.1 and 1 M HCl but degrades at elevated temperature. An *m/z* value of 538.1165 implies the molecular formula [C_20_H_23_N_7_O_7_S_2_ + H]^+^. In comparison with molecular formula of ceftobiprole, one nitrogen atom is replaced with OH group. The fragment ions 520 and 502 (the former signal very intensive) can be explained by the consecutive losses of water molecules, demonstrating the presence of two hydroxyl groups in the molecule of ADP-0 (see Figure S1E). Then, the fragment ion 502 loses CO_2_ and CO in sequence, forming the fragment ions 458 and 430, respectively. Both have been detected and interpreted thus far in the fragmentation spectrum of ceftobiprole. Based on these results, a structural formula was proposed in which the oxime group was first hydrolysed to a keto group and then hydrated to a geminal diol under the influence of the vicinal carbonyl group of the amide. A similar effect can be seen with the ninhydrin molecule, which contains a stable geminal diol group placed between two carbonyl groups. In addition, it has been reported that the carbonyl group of pyruvamide, adjacent to the carbonyl group of the amide, undergoes reversible hydration to form a geminal diol [27].

**Figure 11:**
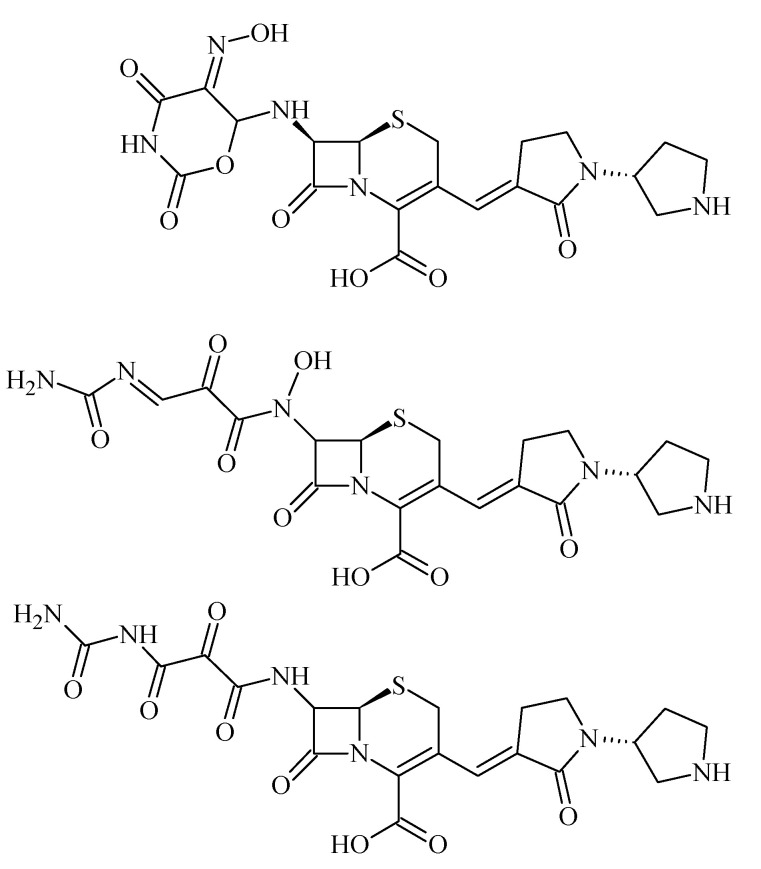
Possible structural formulas proposed for ADP-2.

ADP-1 and ADP-2, each eluting in two peaks, have the molecular masses different by 1 Da. The *m/z* values of 506.1439 and 507.1297 correspond to formulas [C_20_H_23_N_7_O_7_S + H]^+^ and [C_20_H_22_N_6_O_8_S + H]^+^, respectively. In the former, an OH group has been added, while an S and N atom have been removed compared to the ceftobiprole molecule. The simultaneous deletion of a sulphur and nitrogen atom is possible only in the aminothiadiazole ring (if it occurred in the cephem moiety, the molecule would break into two parts, as in the case of ADP-5). However, the signal intensity of the ADP-1 fragmentation mass spectrum was too low to obtain reliable information about the fragmentation, and therefore, only the molecular formula can be provided. Several proposals for the structure of ADP-1, based solely on its molecular formula and the structure of the ceftobiprole molecule, are given in Figure S2. In ADP-2, two atoms of oxygen were added, while N_2_S was removed relative to the ceftobiprole molecule. Again, it can be postulated that the reaction takes place in the aminothiadiazole ring, which contains sulphur and three nitrogen atoms. The fragment ions of *m/z* 308 and below are the same as for ceftobiprole, which implies that the right-hand part of the molecule remains intact. The fragment ions have been successfully interpreted in terms of their molecular formulas (as shown in Table 5); however, several different structural formulas can be proposed which fit the fragmentation pattern comparatively well (see Figure 11).

ADP-3, present in the form of two isomers of *m/z* 479.1348 and 479.1342, can be described as [C_19_H_22_N_6_O_7_S + H]^+^, which in turns equals ADP-2 minus the CO moiety. The fragment ions of *m/z* 365 and below are the same as in the case of ceftobiprole (Figure S1F), which again indicates that the reaction takes place in the aminothiadiazole ring.

ADP-4, a major ADP, is a single isomer of *m/z* 365.1274 with the molecular formula [C_16_H_20_N_4_O_4_S + H]^+^; thus, it is probably a product of the acidic hydrolysis of the amide group attached to the β-lactam ring at the C7 position. The numerous fragment ions have been successfully interpreted based on this proposal of the ADP-4 structure (Figure S1G). As a result of the acid-catalysed hydrolysis of the amide bond, the ceftobiprole molecule breaks down into two parts, ADP-4, and another part that is not detected by either the UV or MS detector. It can be assumed that this second part has neither a chromophore nor an easily ionisable moiety, or it breaks down into even smaller parts.

ADP-5 is a single isomer of *m/z* 265.1178, which can be interpreted as [C_13_H_16_N_2_O_4_ + H]^+^. The main fragment ions (*m/z* 221 and 193) result from the consecutive loss of CO_2_ and CO molecules (Figure S1H). The proposed structural formula assumes that the reaction takes place in the dihydrothiazine ring, with the C4-N5 and C2-S1 bonds breaking and an oxygen atom being attached. ADP-5 has therefore an analogous structure to the DP of ceftaroline fosamil CFI-C, which is formed in a solution stored at room temperature for 29 days [2].

**Scheme 2:**
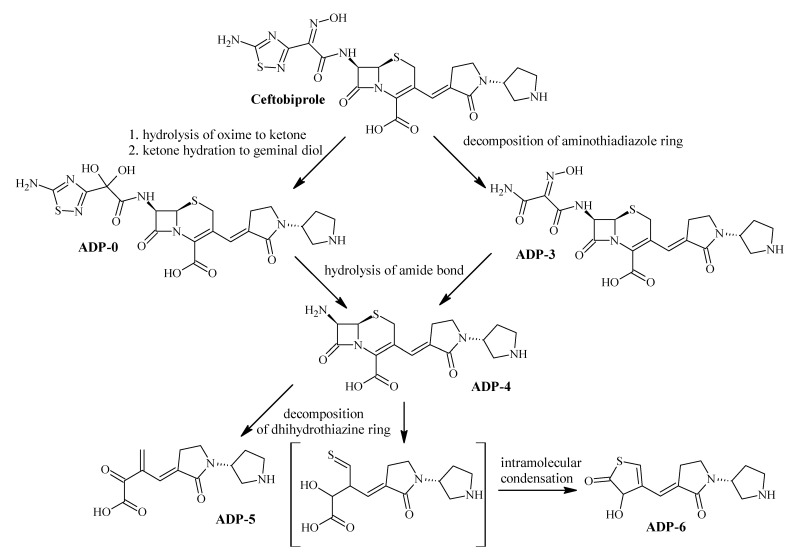
A proposed degradation pathway under acidic conditions.

ADP-6, eluting as the most non-polar and second major ADP, has *m/z* 281.0950, which can be expressed in terms of the molecular formula [C_13_H_16_N_2_O_3_S + H]^+^. Compared to ADP-5, it has a sulphur atom instead of an oxygen atom, and cleavage of the dihydrothiazine ring was therefore proposed, similar to ADP-5 but with the C6-S1 bond breaking. The intermediate product formed would contain a thial (thioaldehyde) group, which is unstable and reactive. Consequently, intramolecular condensation was proposed as the next step of acidic hydrolysis, leading to the formation of a five-member thioester ring (as shown in Scheme 2). This proposal has the following explanation based on the fragmentation spectrum (Figure S1I): (i) there is no loss of CO_2_, which implies the absence of a free carboxyl group, (ii) the strongest signal results from the loss of H_2_O, suggesting the presence of a hydroxyl group in ADP-6, (iii) there are observed transitions corresponding to the losses of CO and H_2_S.

The analysis of the proposed ADP structures explains their high response at 320 nm, in terms of the length of the system of conjugated double bonds. ADPs 1–4 contain four conjugated double bonds, whereas ADP-5 possesses five. The structure of ADP-6 includes an aromatic system consisting of three conjugated bonds and another double bond, separated by a sulphur atom containing lone/single electron pairs. The elongation of the system of conjugated double bonds can serve to explain why the maximum absorption of ADP-5 and ADP-6 is shifted towards higher wavelengths.

On the basis of five unambiguously proposed structures of ADPs, marked with numbers 0, 3, 4, 5 and 6, as well as kinetic data presented in Figure 3, showing the order in which ADPs may be formed, a degradation pathway under acidic conditions was proposed (Scheme 2).

#### 2.4.3. Identification of BDPs

BDP-1 elutes in two peaks of great absorbance at 320 nm and comparably low intensity using the mass detector. Their *m/z*, 553.1279 (a) and 553.1284 (b), are 18 Da greater than the *m/z* of ceftobiprole and can be approximated by the formula [C_20_H_24_N_8_O_7_S_2_ + H]^+^, which indicates the addition of a water molecule to the ceftobiprole molecule. Alkaline hydrolysis is most probable for the amide group in the four-member β-lactam ring, which is highly strained [28]. The two resultant diastereoisomers therefore contain two carboxylic groups, which are observed in the fragmentation mass spectra (Figure S1J) as two consecutive losses of CO_2_ molecules.

There is another lactam group in the ceftobiprole molecule, namely γ-lactam in the 2-pyrrolidone ring. However, the alkaline hydrolysis of this group was not considered for several reasons. Firstly, it is part of a significantly more stable five-member ring and should not hydrolyse easily [28]. Secondly, the C=O bond in this amide group belongs to a system of four conjugated double bonds. Furthermore, the fragment ions of *m/z* 308, 264 and 220, which contain the 2-pyrrolidone ring, are the same as for ceftobiprole, showing that the 2-pyrrolidone ring remains intact in both BDP-1 isomers.

BDP-2, of *m/z* 535.1174, is an isomer of ceftobiprole. It was therefore assumed that the alkaline environment catalyses an intramolecular rearrangement. A lacton structure was proposed, analogous to the DPs of other cephalosporin antibiotics such as cefixime [29] and cefprozil [30], among others, whose structures also contain a double bond in the α, β-position relative to the C3 atom in the dihydrothiazine ring. The absence of the *m/z* 491 ion (Figure S1K) and the extremely low intensity at *m/z* 321 (two fragment ions present in the ceftobiprole fragmentation pathway, formed by the loss of CO_2_ from their parent structures) show that the loss of CO_2_ from BDP-2 is more difficult than from ceftobiprole, and the existence of the lacton group instead of a free carboxyl group may serve as a possible explanation.

Additionally, small chromatographic peaks with retention times above 17 min were identified as BDPs of SBPs. Briefly, four peaks with *m/z* ca. 609.190 were interpreted as “SBP-2 + H_2_O”, while two peaks of *m/z* ca. 719.205 were identified as “SBP-3 + H_2_O”.

#### 2.4.4. Identification of ODPs

In the chromatogram of ODPs of ceftobiprole, there are two main peaks of *m/z* of about 551.112, which correspond to [C_20_H_22_N_8_O_7_S_2_ + H]^+^, i.e., a ceftobiprole molecule with one attached oxygen atom. According to the literature [28,31], there are many reactions of oxidation in which one oxygen atom can be attached to the parent molecule; therefore, the identification of ODPs proved to be very complex. In the case of ceftobiprole, the following reactions with an oxidising agent can be considered: (i) the oxidation of a sulphide (thioether) to a sulphoxide, (ii) the oxidation of a secondary amine to a hydroxylamine, (iii) the oxidation of a tertiary amine to an *N*-oxide, and (iv) the oxidation of a double bond with a formation of an epoxide [28,31]. However, the last two reactions can be disregarded based on the fragmentation mass spectra. The fragment ions 291 and 247 (present for both ODP-1 and ODP-2) are the same as for ceftobiprole, showing that the right-hand part of the molecule (the substituent at C3, containing 2-pyrrolidone and pyrrolidine rings) remains intact. Furthermore, the oxidation of a secondary amine group attached in the position C7 with the formation of a hydroxylamine cannot be easily justified with the use of fragmentation spectra due to the absence of strong signals of fragment ions at *m/z* 308 and 264. In the fragmentation patterns of both ODP-1 and ODP-2, the same transition 489 → 319 can be observed, with a loss of a fragment of mass 169.99 Da. The same loss can be observed in the fragmentation pattern of ceftobiprole, between *m/z* 535 and 365, demonstrating that this fragment relates to the left-hand part of the molecule (the substituent attached to secondary amine at C7, containing the aminothiadiazole ring). Since the substituents at both C7 and C3 proved to remain unchanged during the oxidation, one should consider the reactions taking place in the cephem group, i.e., in the most labile part of a molecule.

The most straightforward reaction is attaching the oxygen atom to the sulphur atom in the dihydrothiazine ring. This process introduces a new chiral centre to the molecule, as the sulphoxides are chiral [28]. For instance, two isomers of cefradine sulphoxide are specified in the European Pharmacopeia monograph as impurities C and D of cefradine [32]. However, it seems to be unlikely that ODP-1 and ODP-2 are geometrical isomers of ceftobiprole sulphoxide. Firstly, the huge difference in retention times (7.1 vs. 10.2 min) is rather untypical for diastereoisomers. Secondly, there are considerable differences between their fragmentation spectra: the fragment ions of *m/z* 507, 463 and 417 have a significant intensity for ODP-2 but are hardly distinguishable from noise for ODP-1. Consequently, two different structural formulas have been proposed for ODP-1 and ODP-2.

ODP-1 can be thought of as a sulphoxide of ceftobiprole (see Figure S1L). The most convincing evidence of this statement is the transition 489 → 441 with the loss of sulphoxide group (SO). Some other transitions in the fragmentation mass spectrum can be explained as the counterparts of the corresponding transitions observed for the ceftobiprole molecule: 551 → 489 with the loss of CO_2_ and H_2_O, similar to 535 → 473 for ceftobiprole, and 489 → 319, which relates to the loss of the fragment of mas 170 Da, discussed earlier.

ODP-2 has a fragmentation pattern with a greater number of peaks compared to ODP-1 (Figure S1M). The transitions 551 → 507 → 463 correspond to two consecutive losses of carbon dioxide, which indicate the presence of two carboxyl groups. This is very similar to the fragmentation mass spectrum of BDP-1, in which the similar cascade 553 → 509 → 465 was observed. Similarly to ODP-1, the fragment ions 489 and 441 are present and can be interpreted using the same molecular formulas, but to the best of our knowledge, they relate to different structural formulas. A structure of ODP-2 has been proposed which can be best explained with use of fragmentation mass spectrum but should be analysed with caution.

Additionally, chromatographic peaks with retention times above 17 min were identified as ODPs of SBPs. Four peaks with *m/z* ca. 607.175 were interpreted as “SBP-2 + O”, while two peaks of *m/z* ca. 717.190 were identified as “SBP-3 + O”. They are not likely to form under long storage of ceftobiprole; hence, they will not be discussed in detail.

#### 2.4.5. Identification of PDPs and TDPs

The isomerisation of ceftobiprole occurs as a result of photolytic degradation, leading to the formation of two new DPs, PDP-1a and PDP-1c, as well as an increase in the content of PDP-1b, which is also present in the non-degraded solution of ceftobiprole. During thermolytic degradation, another isomer of ceftobiprole forms, labelled with the acronym TDP-1. These DPs possess the same monoisotopic mass as ceftobiprole and have a very similar fragmentation pattern. Due to the technical limitations of MS, the exact structures of the isomers of ceftobiprole cannot be elucidated. There are many possible reactions that could take place, including racemisation, epimerisation, ring transformation or migration of a double bond in a dihydrothiazine ring from the Δ^3^ position (between C3 and C4) to the Δ^2^ position (between C2 and C3) [28].

PDP-2, with *m/z* 503.1447, can be interpreted as [C_20_H_22_N_8_O_6_S + H]^+^, i.e., the ceftobiprole molecule without one sulphur atom. However, due to the very low intensity (both in UV and MS) and low quality of the fragmentation mass spectrum, the exact structure of PDP-2 could not be determined. Some tentative proposals for the structure of PDP-2 are given in Figure S3.

#### 2.4.6. Identification of ODPs Formed in Solid-State Degradation

As can be seen in Table 4, solid-state degradation carried out for 21 days resulted in the formation of very small quantities of individual DPs (less than 0.7% in all cases). Consequently, it was impossible to record high-quality fragmentation mass spectra and thus to determine the structures of these DPs. Hence, only the molecular formulas of the DPs characteristic of solid-state degradation could be proposed. ODP-S1, S2 and S3, with *m/z* 551.1, are isomers of ODP-1 and ODP-2 with the molecular formula [C_20_H_22_N_8_O_7_S_2_ + H]^+^. This means that one oxygen atom is attached to the ceftobiprole molecule, but due to the low intensity of the fragmentation spectrum, it is not possible to determine unambiguously where this atom has been attached. In the mass spectrum of ODP-S4, the peak of highest intensity was located at *m/z* 550.1035, and the neighbouring peaks of its isotopic pattern were separated by 0.5. It can be interpreted that this peak corresponds to the ion [M + 2H]^2+^, and then, the peak at *m/z* 569.0810 observed in the same spectrum can be assigned to [M + H + K]^2+^. Based on these results, a dimeric product of ceftobiprole of molecular formula C_40_H_42_N_16_O_14_S_4_ can be proposed. This formula corresponds to two ceftobiprole molecules plus two oxygen atoms minus two hydrogen atoms. Indeed, [C_40_H_42_N_16_O_14_S_4_ + 2H]^2+^ has a theoretical *m/z* of 550.1047, which differs from the experimental value by 2.2 ppm, i.e., it lies in the allowable deviation range (<5 ppm).

## 3. Materials and Methods

### 3.1. Materials

Ceftobiprole was obtained from Basilea Pharmaceutica International Ltd. (Basel, Switzerland), manufactured on March 2017 and stored in a freezer at −20 °C). Deionised water was obtained from a Labconco System by Millipore (Bedford, MA, USA). Acetonitrile (ACN, gradient grade for HPLC, ≥99.9%), ACN for LC-MS (≥99.9%), methanol (MeOH, gradient grade for HPLC, ≥99.9%) and DMSO (for HPLC, 99.7%) were obtained from Honeywell (Charlotte, NC, USA). Sodium hydroxide (≥98.8%), ammonium hydrogencarbonate (≥99.0%) and ammonium formate (≥99.0%) were delivered by Chempur (Piekary Śląskie, Poland); HCl (pure p.a., 35–38%) and H_2_O_2_ (pure p.a., 30%) were bought from POCH (Gliwice, Poland); acetic acid (for LC-MS, 99.8%) and ammonium acetate (reag. Ph. Eur., ≥98.0%) were purchased from Merck (Darmstadt, Germany); acetic acid (pharma grade, 99.9%) was obtained from AppliChem (Darmstadt, Germany); and ammonium acetate (for LC-MS, ≥99.0%) and formic acid (reag. Ph. Eur., ≥98%) were manufactured by Sigma-Aldrich (Darmstadt, Germany).

The following chromatographic columns were used: Kinetex C18 (150 × 3 mm, 2.6 μm), Kinetex PS C18 (150 × 2.1 mm, 2.6 μm) and Kinetex Biphenyl (150 × 2.1 mm; 1.7 μm) from Phenomenex (Torrance, CA, USA), as well as Accucore AQ C18 (150 × 4.6 mm, 2.6 μm) from Thermo Fisher Scientific (Waltham, MA, USA).

### 3.2. Solution-State Degradation Study

#### 3.2.1. Acidic Hydrolysis with Temperature

For this study, 3 mg of ceftobiprole was dissolved in 3 mL of 1 M HCl. The process was carried out in a stoppered volumetric flask in a water bath at 50, 60 or 70 °C, and incubated for 0, 90, 180, 270 and 360 min (*n* = 5).

#### 3.2.2. Alkaline Hydrolysis

For this study, 1 mg of ceftobiprole was dissolved in 1 mL of 0.01 M NaOH in a HPLC vial and immediately placed in the autosampler at 25 °C; 1 µL of the solution was injected at specified time intervals (45 min or its multiple) for 720 min (12 h, *n* = 13).

#### 3.2.3. Oxidative Degradation

For this study, 1 mg of ceftobiprole was dissolved in 0.1 mL DMSO or 1 M HCl and sonicated until complete dissolution. Then, 0.8 mL of water and 0.1 mL of 30% H_2_O_2_ solution was added, equating to 1 mg/mL ceftobiprole in 3% H_2_O_2_ in 10% DMSO or 0.1 M HCl. Samples were placed in in the autosampler at 25 °C and incubated for 1080 min (18 h); 1 µL of each solution was injected every 90 min (*n* = 13).

#### 3.2.4. Photolytic Degradation

For this study, 3 mg of ceftobiprole was dissolved in 0.3 mL DMSO or 1 M HCl, the sample was vortexed or sonicated until dissolved, and then, 2.7 mL of water was added. The solution was irradiated with UV light at 366 and 254 nm at room temperature in an open flask, and samples were taken after 0, 90, 180, 270, 360 and 1410 min (23.5 h, *n* = 6).

#### 3.2.5. Thermolytic Degradation

For this study, 3 mg of ceftobiprole was dissolved in 0.3 mL DMSO, sonicated, and then, 2.7 mL of water was added. The solution was heated in a stoppered volumetric flask in a water bath at 50, 60 or 70 °C, and the samples were incubated for 0, 90, 180, 270, 360 and 1410 min (23.5 h, *n* = 6).

### 3.3. Solid-State Degradation Study

For the solid-state degradation study, 1 mg of ceftobiprole was weighed into a 1 mL volumetric flask and spread out in a thin layer on the bottom of the flask. The samples were placed under the following conditions:

- At 50 and 60 °C for 7 and 21 days without control of humidity (Memmert oven, Memmert, GmbH + Co. KG, Schwabach FRG, Germany);
- At 50 ± 2 °C/75 ± 5% RH (AtomControl Memmert, GmbH + Co. KG, Schwabach FRG, Germany), tested at seven-day intervals over a period of 21 days;
- Under illumination with UV at 366 nm at room temperature, tested at seven-day intervals over a period of 21 days;
- In a chamber simulating natural sunlight at an intensity of 5260 ± 316 lx for 10 and 21 days, at room temperature.

Finally, samples were dissolved in 0.1 mL DMSO, filled up to the mark with water and analysed.

### 3.4. HPLC/UV Analysis

The HPLC analysis was performed using a Shimadzu Nexera-i LC-2040C Plus (Kioto, Japan) liquid chromatograph with UV–VIS detector, equipped with LabSolutions 5.92 data processing software. In order to optimise the method, the following 0.05 M buffers were prepared as mobile phase A: pH 7.8—by dissolving ammonium hydrogencarbonate in water, pH 6.8—by dissolving ammonium hydrogencarbonate in water and adjusting the pH with acetic acid, pH 5.8 and 4.8—by dissolving ammonium acetate in water and adjusting the pH with acetic acid, pH 3.8 and 2.8—by dissolving ammonium formate in water and adjusting the pH with formic acid.

### 3.5. LC/MS/MS Analysis

Identifications were performed using an Dionex UltiMate 3000 UPLC system (Thermo Fisher Scientific, USA) connected to a high resolution maXis 4G Q-TOF mass detector (Bruker Daltonik, Germany) and controlled by Bruker HyStar 3.2 SR2 software. The MS was equipped with an ESI ion source and operated in the positive ion mode, in the full scan range from *m/z* 100 to 1500 with fragmentation of the five most intensive signals. The following MS conditions were used: capillary voltage 3800 V, endplate offset −500 V, collision cell RF voltage 350.0 Vpp, nebuliser gas pressure 1.6 bar, dry heater temperature 200 °C and dry gas flow rate 8 L/min. In MS/MS mode, the collision energy was ramped linearly, depending on the *m/z*. For *m/z* 200, 400 and 800, the voltage was set to 20, 30 and 40 eV, respectively. Nitrogen was used as a dry and collision gas. The MS was calibrated using a sodium formate solution prior to each injection. Data processing was performed using Bruker DataAnalysis 4.0 software. Molecular formulas were calculated with mass accuracy (expressed as error) below 5 ppm and isotopic pattern (expressed as mSigma) generally below 30. mSigma (milliSigma) is a rate for the agreement of the theoretical and measured isotopic pattern of the mass peak of interest. It combines the standard deviation of the masses and intensities for all isotopic peaks. The lower the mSigma, the better the fit.

## 4. Conclusions

A novel chromatographic method was developed for the simultaneous separation of ceftobiprole from its SBPs and DPs.

The susceptibility of ceftobiprole to degradation under various stress conditions has been ordered based on the determined rate constants as follows: in 0.1 M HCl (1.31 × 10^−6^ s^−1^), in 10% DMSO under photolysis at 254 nm (4.28 × 10^−6^ s^−1^) and at 366 nm (3.03 × 10^−5^ s^−1^), in a 3% aqueous solution of H_2_O_2_ (7.07 × 10^−5^ s^−1^) and then in 0.01 NaOH (7.08 × 10^−4^ s^−1^). The degradation in 0.1 M NaOH was too fast to accurately determine the rate constant. Activation energies were calculated using an Arrhenius plot for the thermolytic degradation performed in 10% DMSO and 1 M HCl at 50, 60 and 70 °C; hence, it was possible to determine low values of rate constants at room temperature by the extrapolation of these plots. The degradation rate constant of ceftobiprole in 10% DMSO at 25 °C was 9.74 × 10^−8^ s^−1^, which means that 10% of the initial API content would undergo degradation in 12.5 days.

In the case of degradation in the solid state, the determined rate constants were several orders of magnitude lower than those obtained for the degradation in solution.

The structural formulas of SBPs and DPs of ceftobiprole were proposed for the first time, based on MS/MS spectra. Four novel structural formulas for SBPs were proposed. With reference to the reported synthetic route, the formation of SBPs can take place inside reactions during the deprotection of carboxylic and oxime groups. One major ADP was formed in 0.1 M HCl at room temperature, and six another ADPs were formed in 1 M HCl at elevated temperature, three of them as pairs of isomers. It was possible to propose structural formulas for five ADPs, but only the molecular formula could be proposed for the two remaining ADPs. Based on the structure of the ADPs, it can be hypothesised that HCl mainly attacks the sulphur and nitrogen atoms of the ceftobiprole molecule, mainly in the aminothiadiazole and cephem moieties. In alkaline hydrolysis, two isomers of the open-ring ceftobiprole and a lactone were formed as the major DPs. Two predominant DPs were generated in the reaction of ceftobiprole with 3% H_2_O_2_, regardless of whether 10% DMSO or 0.1 M HCl was used as the solvent. UV irradiation caused the isomerisation of ceftobiprole, but the structure of the three resultant isomers could not be determined due to the limitations of MS. The same applies to the ceftobiprole isomer formed during heating at 70 °C. In the MS analysis, thirteen new structural formulas of SBPs and DPs were proposed, five relating to pairs of isomers, while molecular formulas were determined for three other compounds obtained in solution as well as four ODPs characteristic of solid-state degradation.

## Figures and Tables

**Table 1:** The elaborated optimal chromatographic conditions for the separation of ceftobiprole and its related substances.

Column	Kinetex Biphenyl 150 × 2.1 mm; 1.7 μm	
Column temperature	40 °C	
Autosampler temperature	4 °C—for method optimization 25 °C—for kinetic studies	
Mobile phase	A: 20 mM ammonium acetate buffer, pH = 5.8 B: ACN	
Flow rate	0.3 mL/min	
Gradient	Time (min)	%B
	0	0
	15	10
	28	90
	33	90
	33.1	0
	45	0
Injection volume	1 μL—for kinetic studies 20 µL—for LC/MS studies	
Detection wavelength	320 nm	

**Table 2:** Comparison of ceftobiprole stability in 10% DMSO and 0.1 M HCl solution. The value of 100% refers to the initial peak area of ceftobiprole without synthesis by-products (SBPs) and degradation products (DPs).

Time Point	10% DMSO	0.1 M HCl
	% Drug Remaining	% Drug Remaining
0	100.0%	100.0%
3 h	99.5%	98.0%
6 h	99.4%	96.9%
12 h	99.2%	94.3%
24 h	98.9%	89.6%

**Table 3:** Experimental stress conditions in solution-state studies (solvents, temperatures and/or irradiation wavelengths), the percentage of DPs formed after 6 h of degradation, and the calculated degradation rate constants with R^2^.

Degradation Conditions		Solvent	DPs Formed after 6 h	k (s^−1^)	t_90_ (min)	R^2^	E_a_ (kJ/mol)
Acidic hydrolysis	25 °C	0.1 M HCl	ADP-0 (0.94%)	1.31 × 10^−6^	1340.2	0.9899	na
	25 °C	1 M HCl	The rate constant is determined from the extrapolation of Arrhenius plot	1.77 × 10^−6^	993.8	na	102.3
	50 °C		ADP-1a (0.95%), ADP-1b (1.03%) ADP-2a (2.48%), ADP-2b (2.59%), ADP-4 (11.10%)	4.34 × 10^−5^	40.5	0.9999	
	60 °C		ADP-1a (1.30%), ADP-1b (1.42%) ADP-2a (5.18%), ADP-2b (5.52%), ADP-4 (9.13%), ADP-5 (0.81%), ADP-6 (1.69%)	1.33 × 10^−4^	13.2	0.9972	
	70 °C		ADP-1a (0.44%), ADP-1b (0.48%) ADP-2a (1.42%), ADP-2b (1.79%), ADP-3a (0.25%), ADP-4 (2.86%), ADP-3b (0.23%), ADP-5 (1.11%), ADP-6 (2.20%)	3.99 × 10^−4^	4.4	0.9796	
Alkaline hydrolysis	25 °C	0.01 M NaOH	BDP-1a (32.28%), BDP-1b (20.39%), BDP-2 (2.70%)	1.78 × 10^−4^	9.8	0.9909	na
Oxidation	3% H_2_O_2_	10% DMSO	ODP-1 (7.83%), ODP-2 (27.33%)	2.56 × 10^−5^	68.7	0.9991	na
		0.1 M HCl	ODP-1 (20.91%), ODP-2 (58.12%)	1.33 × 10^−4^	13.2	0.9932	na
Photolysis	254 nm	10% DMSO	PDP-1a (3.03%), PDP-1b * (1.26%)	4.28 × 10^−6^	410.0	0.9991	na
		0.1 M HCl	PDP-1a (1.68%), PDP-1b * (1.90%), PDP-2 (0.16%), PDP-1c (0.09%)	4.72 × 10^−6^	372.3	0.9843	na
	366 nm	10% DMSO	PDP-1a (15.48%), PDP-1b * (0.26%), PDP-1c (0.05%)	3.03 × 10^−5^	58.0	0.9896	na
		0.1 M HCl	PDP-1a (10.61%), PDP-1b * (0.19%), PDP-2 (0.15%), PDP-1c (0.15%)	3.43 × 10^−5^	51.2	0.9851	na
Thermolysis	25 °C	10% DMSO	The rate constant is determined from the extrapolation of Arrhenius plot	9.74 × 10^−8^	18,023.1	na	106.3
	50 °C		BDP-1b (0.45%)	2.62 × 10^−6^	669.4	0.9837	
	60 °C		BDP-1b (1.32%), PDP-1b * (0.31%), TDP-1 (0.15%)	9.25 × 10^−6^	189.9	0.9983	
	70 °C		BDP-1b (1.32%), ADP-5 (0.35%), PDP-1b * (0.60%), TDP-1 (0.83%)	2.63 × 10^−5^	66.9	0.9962	

* The initial content of PDP-1b in the non-degraded sample of ceftobiprole is 0.25%. “na” = “not applicable”.

**Table 4:** Results of solid-state degradation of ceftobiprole.

Condition	Exposure	DPs Detected	k (s^−1^)	t_90_ (d)	R^2^
thermal	21 d at 50 °C/uncontrolled RH	BDP-1b (7 d: 0.20%, 21 d: 0.15%), ODP-2 (7 d: 0.10%, 21 d: 0.12%), PDP-1b (7 d: 0.33%, 21 d: 0.32%)	6.92 × 10^−9^	176.2	0.9949
	21 d at 60 °C/uncontrolled RH	BDP-1b (7 d: 0.25%, 21 d: 0.16%), ODP-2 (7 d: 0.10%, 21 d: 0.12%), PDP-1b (7 d: 0.39%, 21 d: 0.34%)	9.34 × 10^−9^	130.5	0.9842
thermal, high humidity	21 d at 50 °C/75% RH	BDP-1b (7 d: 0.21%, 14 d: 0.40%, 21 d: 0.30%), ODP-2 (7 d: 0.06%, 14 d: 0.07%, 21 d: 0.07%), PDP-1b (7 d: 0.31%, 14 d: 0.27%, 21 d: 0.30%)	3.61 × 10^−9^	337.7	0.9894
photolysis, VIS	1.26–2.65 million lux-hours	ODP-1 (10 d: 0.33%, 21 d: 0.64%), ODP-S1 (10 d: 0.06%, 21 d: 0.14%), ODP-S2 (10 d: 0.33%, 21 d: 0.65%), ODP-2 (10 d: 0.30%, 21 d: 0.51%), ODP-S3 (10 d: 0.23%, 21 d: 0.43%), PDP-1b (10 d: 0.39%, 21 d: 0.49%), ODP-S4 (10 d: 0.43%, 21 d: 0.69%)	1.68 × 10^−7^	7.2	0.8415
photolysis, UV	21 d, 366 nm	ODP-1 (7 d: 0.26%, 14 d: 0.33%, 21 d: 0.45%), ODP-S1 (7 d: 0.06%, 14 d: 0.05%, 21 d: 0.07%), ODP-S2 (7 d: 0.28%, 14 d: 0.23%, 0.32%), ODP-2 (7 d: 0.25%, 14 d: 0.29%, 21 d: 0.37%), ODP-S3 (7 d: 0.19%, 14 d: 0.15%, 21 d: 0.22%), PDP-1b (7 d: 0.40%, 14 d: 0.34%, 21 d: 0.36%), ODP-S4 (7 d: 0.02%, 14 d: 0.32%, 21 d: 0.33%)	2.60 × 10^−8^	46.9	0.9893

**Table 5:** Summary of mass spectrometric data for ceftobiprole and its SBPs and DPs.

**Name**	RRT	Experimental *m/z*	Proposed Molecular Formula of [M + H]^+^	Error (ppm)	mSigma	Experimental *m/z* of Major Fragment Ions	Proposed [M + H]^+^ Formula of Fragment Ions	Proposed Structure
Ceftobiprole	1.00	535.1167	C_20_H_23_N_8_O_6_S_2_	1.8	26.3	473.1182 365.1264 321.1372 308.1063 291.1207 264.1168 220.1435 203.1179	C_19_H_21_N_8_O_3_S_2_ C_16_H_21_N_4_O_4_S C_15_H_21_N_4_O_2_S C_14_H_18_N_3_O_3_S C_14_H_17_N_3_O_4_ C_13_H_18_N_3_OS C_12_H_18_N_3_O C_12_H_15_N_2_O	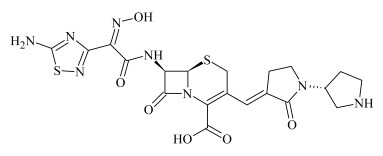
SBP-1	1.42	567.1431	C_21_H_27_N_8_O_7_S_2_	1.4	6.3	523.1537 489.1660 370.1519 319.1763 264.1171 235.0907 220.1445 202.1335	C_20_H_27_N_8_O_5_S_2_ C_20_H_25_N_8_O_5_S C_18_H_20_N_5_O_4_ C_16_H_23_N_4_O_3_ C_13_H_18_N_3_OS C_12_H_15_N_2_OS C_12_H_18_N_3_O C_12_H_16_N_3_	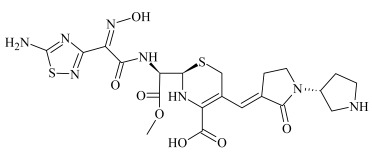
SBP-2a SBP-2b	1.48 1.59	591.1791 591.1799	C_24_H_31_N_8_O_6_S_2_	1.9 0.5	17.6 36.8	535.1176 491.1248 473.1152 365.1288 321.1348 308.1060 291.1206 264.1167 228.0182 220.1430 203.1173	C_20_H_23_N_8_O_6_S_2_ C_19_H_23_N_8_O_4_S_2_ C_19_H_21_N_8_O_3_S_2_ C_16_H_21_N_4_O_4_S C_15_H_21_N_4_O_2_S C_14_H_18_N_3_O_3_S C_14_H_17_N_3_O_4_ C_13_H_18_N_3_OS C_6_H_6_N_5_O_3_S C_12_H_18_N_3_O C_12_H_15_N_2_O	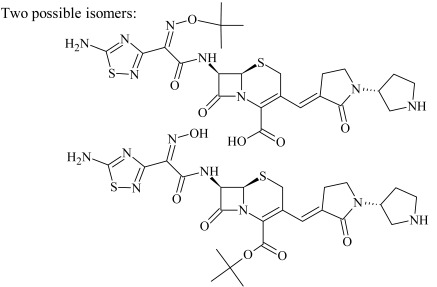
SBP-3a SBP-3b	1.65 1.67	701.1926 701.1933	C_33_H_33_N_8_O_6_S_2_	4.7 3.7	18.6 13.1	394.0962 308.1044 291.1213 264.1164 247.1234 220.1444 203.1180 167.0857	C_21_H_18_N_2_O_4_S C_14_H_18_N_3_O_3_S C_14_H_17_N_3_O_4_ C_13_H_18_N_3_OS C_13_H_17_N_3_O_2_ C_12_H_18_N_3_O C_12_H_15_N_2_O C_13_H_12_	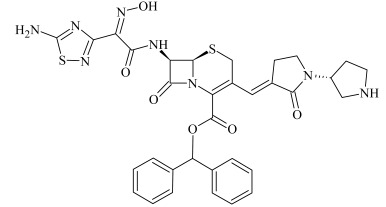
ADP-0		538.1165	C_20_H_24_N_7_O_7_S_2_	1.6	14.8	476.1171 458.1067 448.1201 308.1063 291.1209 264.1164 220.1441 203.1178	C_19_H_22_N_7_O_4_S_2_ C_19_H_20_N_7_O_3_S_2_ C_18_H_22_N_7_O_3_S_2_ C_14_H_18_N_3_O_3_S C_14_H_17_N_3_O_4_ C_13_H_18_N_3_OS C_12_H_18_N_3_O C_12_H_15_N_2_O	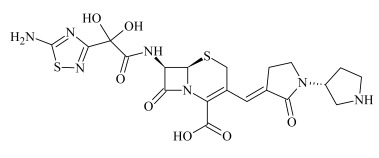
ADP-1a ADP-1b	0.54 0.58	506.1439 506.1459	C_20_H_24_N_7_O_7_S	2.7 −1.2	7.2 80.0	---------	---------------	Due to low intensity of fragmentation mass spectrum, the structural formula cannot be determined. Three tentative structural proposals are shown in Figure S2.
ADP-2a ADP-2b	0.69 0.75	507.1297 507.1303	C_20_H_23_N_6_O_8_S	−1.0 −2.0	12.3 13.5	464.1230 446.1141 291.1201 264.1177 247.1288 220.1424 203.1171 179.0814	C_19_H_22_N_5_O_7_S C_19_H_20_N_5_O_6_S C_14_H_17_N_3_O_4_ C_13_H_18_N_3_OS C_13_H_17_N_3_O_2_ C_12_H_18_N_3_O C_12_H_15_N_2_O C_9_H_11_N_2_O_2_	The structure cannot be unambiguously determined. Many structural formulas can be proposed based on the fragmentation mass spectrum (three examples are shown in Figure 11).
ADP-3a ADP-3b	0.78 0.84	479.1348 479.1342	C_19_H_23_N_6_O_7_S	−1.0 0.4	16.5 125.6	418.1190 365.1334 321.1399 291.1194 264.1172 223.0672 203.1189 179.0820	C_18_H_20_N_5_O_5_S C_16_H_21_N_4_O_4_S C_15_H_21_N_4_O_2_S C_14_H_17_N_3_O_4_ C_13_H_18_N_3_OS C_10_H_12_N_2_O_4_ C_12_H_15_N_2_O C_9_H_11_N_2_O_2_	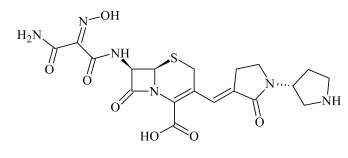
ADP-4	0.81	365.1274	C_16_H_21_N_4_O_4_S	1.0	18.9	293.1406 291.1203 259.1537 232.1437 220.1424 207.0592 203.1168 165.1029	C_14_H_21_N_4_OS C_14_H_17_N_3_O_4_ C_14_H_19_N_4_O C_13_H_18_N_3_O C_12_H_18_N_3_O C_10_H_11_N_2_OS C_12_H_15_N_2_O C_9_H_13_N_2_O	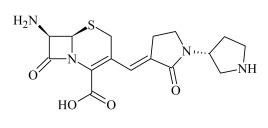
ADP-5	0.88	265.1178	C_13_H_17_N_2_O_4_	1.9	2.8	221.1281 203.1173 193.1312 154.0873 152.0745 137.0610	C_12_H_17_N_2_O_2_ C_12_H_15_N_2_O C_11_H_17_N_2_O C_8_H_12_NO_2_ C_8_H_10_NO_2_ C_8_H_8_O_2_	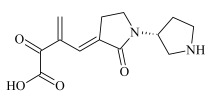
ADP-6	1.16	281.0950	C_13_H_17_N_2_O_3_S	1.7	22.3	263.0846 253.0885 235.0739 219.1124 194.0272 152.0932	C_13_H_15_N_2_O_2_S C_12_H_17_N_2_O_2_S C_12_H_15_N_2_OS C_12_H_15_N_2_O_2_ C_9_H_8_NO_2_S C_8_H_12_N_2_O	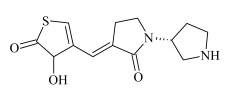
BDP-1a BDP-1b	0.62 0.65	553.1279 553.1284	C_20_H_25_N_8_O_7_S_2_	0.6 −0.4	41.2 35.3	509.1376 465.1485 431.1616 352.1189 308.1058 287.1505 264.1688 220.1428 202.1337	C_19_H_25_N_8_O_5_S_2_ C_18_H_25_N_8_O_3_S_2_ C_18_H_23_N_8_O_3_S C_15_H_20_N_4_O_4_S C_14_H_18_N_3_O_3_S C_15_H_19_N_4_O_2_ C_13_H_18_N_3_OS C_12_H_18_N_3_O C_12_H_16_N_3_	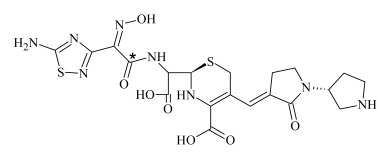
BDP-2	1.18	535.1174	C_20_H_23_N_8_O_6_S_2_	0.5	14.1	308.1066 291.1202 264.1163 246.1062 228.0172 203.1171	C_14_H_18_N_3_O_3_S C_14_H_17_N_3_O_4_ C_13_H_18_N_3_OS C_13_H_16_N_3_S C_6_H_5_N_5_O_3_S C_12_H_15_N_2_O	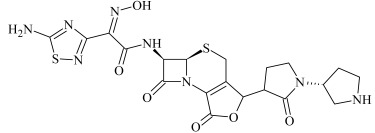
ODP-1	0.53	551.1118	C_20_H_23_N_8_O_7_S_2_	1.3	28.5	489.1130 441.1436 357.0770 319.1211 291.1216 247.1311 220.1429 218.1279	C_19_H_21_N_8_O_4_S_2_ C_19_H_21_N_8_O_3_S C_15_H_13_N_6_O_3_S C_15_H_19_N_4_O_2_S C_14_H_17_N_3_O_4_ C_13_H_17_N_3_O_2_ C_12_H_18_N_3_O C_12_H_16_N_3_O	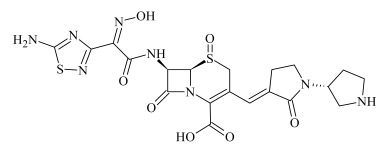
ODP-2	0.77	551.1118	C_20_H_23_N_8_O_7_S_2_	1.4	31.7	507.1217 489.1132 463.1332 441.1458 417.1445 319.1212 291.1220 247.1311 218.1279	C_19_H_23_N_8_O_5_S_2_ C_19_H_21_N_8_O_4_S_2_ C_18_H_23_N_8_O_3_S_2_ C_19_H_21_N_8_O_3_S C_17_H_21_N_8_O_3_S C_15_H_19_N_4_O_2_S C_14_H_17_N_3_O_4_ C_13_H_17_N_3_O_2_ C_12_H_16_N_3_O	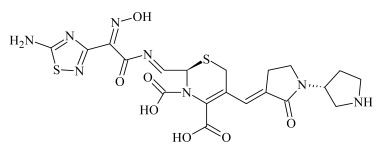
ODP-S1 ODP-S2 ODP-S3	0.59 0.74 0.90	551.1112 551.1123 551.1122	C_20_H_23_N_8_O_7_S_2_	2.4 0.4 0.7	33.0 10.2 15.6	---------	---------------	Due to low intensity of fragmentation mass spectrum, the structural formula cannot be determined.
ODP-S4	1.44	[M + 2H]^2+^ 550.1035	[M + 2H]^2+^ C_40_H_44_N_16_O_14_S_4_	2.2	28.3	---------	---------------	Due to low intensity of fragmentation mass spectrum, the structural formula cannot be determined.
PDP-1a PDP-1b PDP-1c TDP-1	1.06 1.14 1.40 1.19	535.1174 535.1175 535.1178 535.1172	C_20_H_23_N_8_O_6_S_2_	0.4 0.3 −0.2 0.9	34.2 34.3 18.4 16.9	The same as for ceftobiprole		Isomers of ceftobiprole
PDP-2	1.26	503.1447	C_20_H_23_N_8_O_6_S	1.8	20.7	---------	---------------	Due to low intensity of fragmentation mass spectrum, the structural formula cannot be determined. Two tentative structural proposals are shown in Figure S3.

## Data Availability

Not applicable.

## Supplementary Materials

The following supporting information can be downloaded at: https://www.mdpi.com/article/10.3390/ijms232315252/s1.
