# Supplementary material for: Study of Degradation Kinetics and Structural Analysis of Related Substances of Ceftobiprole by HPLC with UV and MS/MS Detection
Source: Int J Mol Sci. 2022 Dec 3;23(23):15252. doi: 10.3390/ijms232315252 (PMC9735983; doi:10.3390/ijms232315252)
Supplement: Supplementary file 1 [file ijms-23-15252-s001.zip › ijms-2050350-supplementary.pdf]

Supplementary Material

Study of Degradation Kinetics and Structural Analysis of Related Substances of Ceftobiprole by HPLC with UV and MS/MS Detection

Dariusz Boczar [1], Katarzyna Bus [2] and Katarzyna Michalska [1,*]

[1] Department of Synthetic Drugs, National Medicines Institute, Chełmska 30/34, 00-725 Warsaw, Poland
[2] Department of Spectrometric Methods, National Medicines Institute, Chełmska 30/34, 00-725 Warsaw, Poland

Table of contents:

Figure S1. Fragmentation mass spectra and proposed fragmentation pathways for:
(A) ceftobiprole, p. 2
(B) SBP-1, p. 3
(C) SBP-2 (common for both isomers), p. 4
(D) SBP-3 (common for both isomers), p. 5
(E) ADP-0, p. 6
(F) ADP-3 (common for both isomers), p. 7
(G) ADP-4, p. 8
(H) ADP-5, p. 9
(I) ADP-6, p. 10
(J) BDP-1 (common for both isomers), p. 11
(K) BDP-2, p. 12
(L) ODP-1, p. 13
(M) ODP-2. p. 14
Figure S2. Proposals of the structure of ADP-1. p.15
Figure S3. Proposals of the structure of PDP-2. p. 15

A. Ceftobiprole

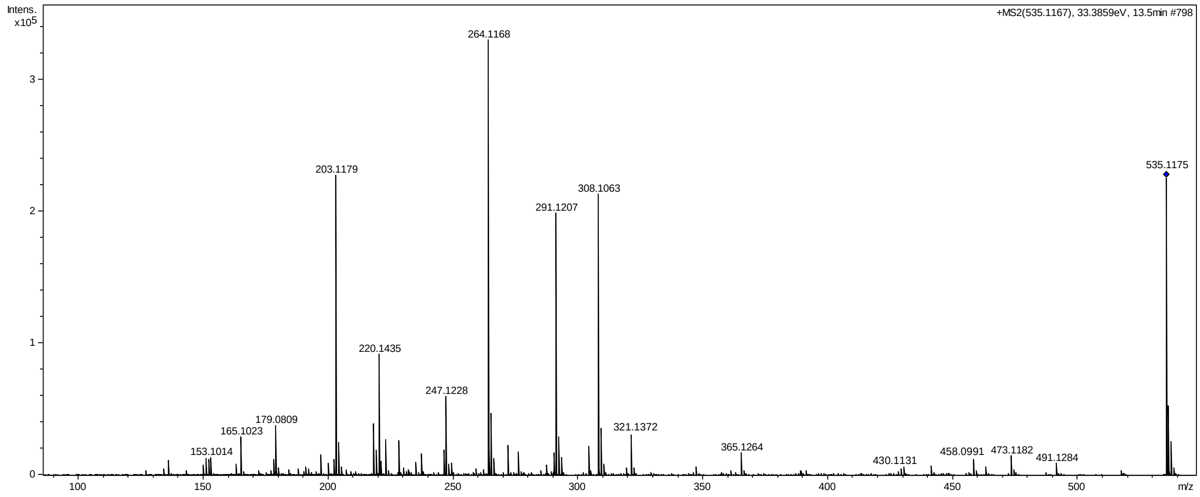

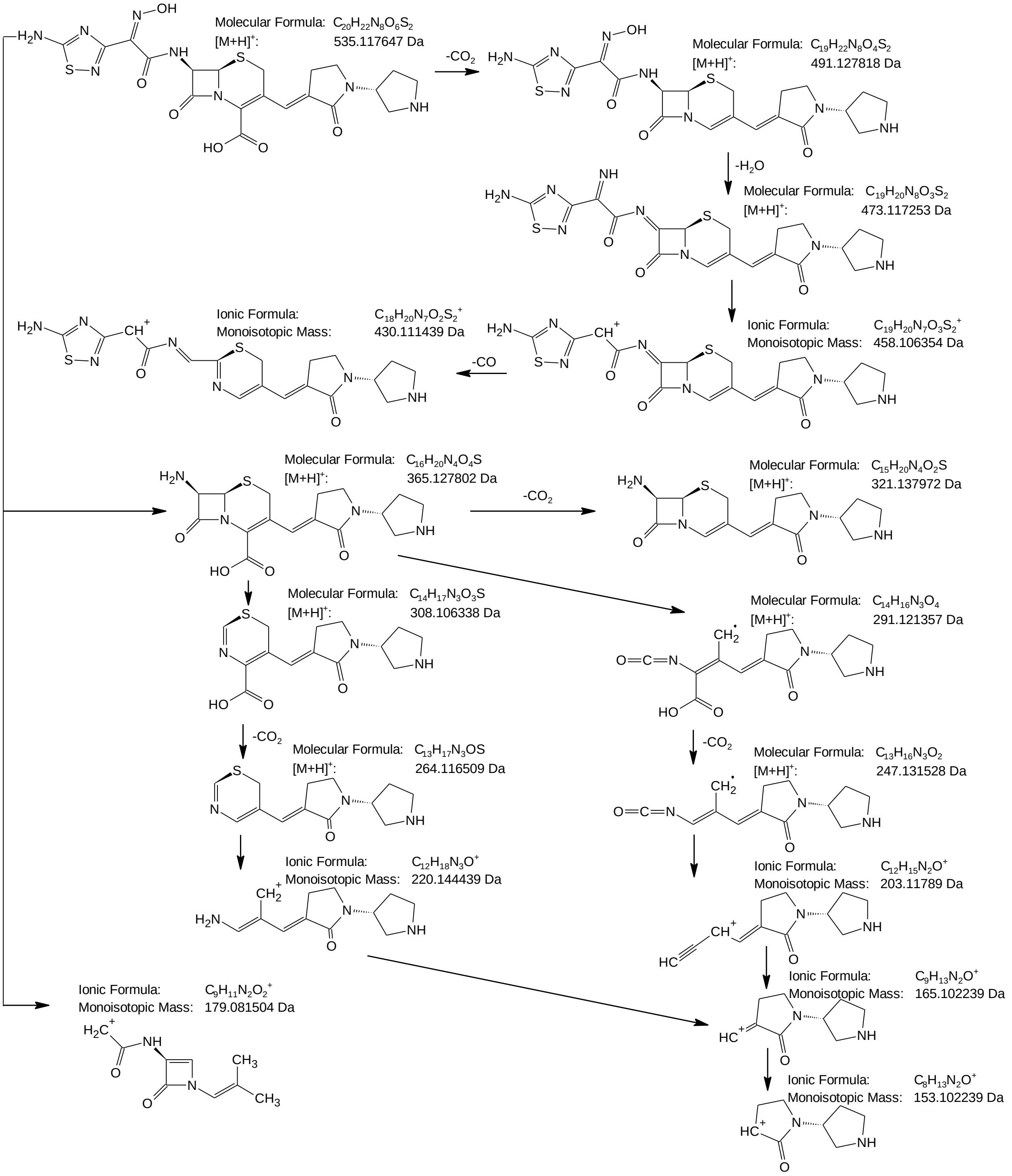

B. SBP-1 (*m/z* 567)

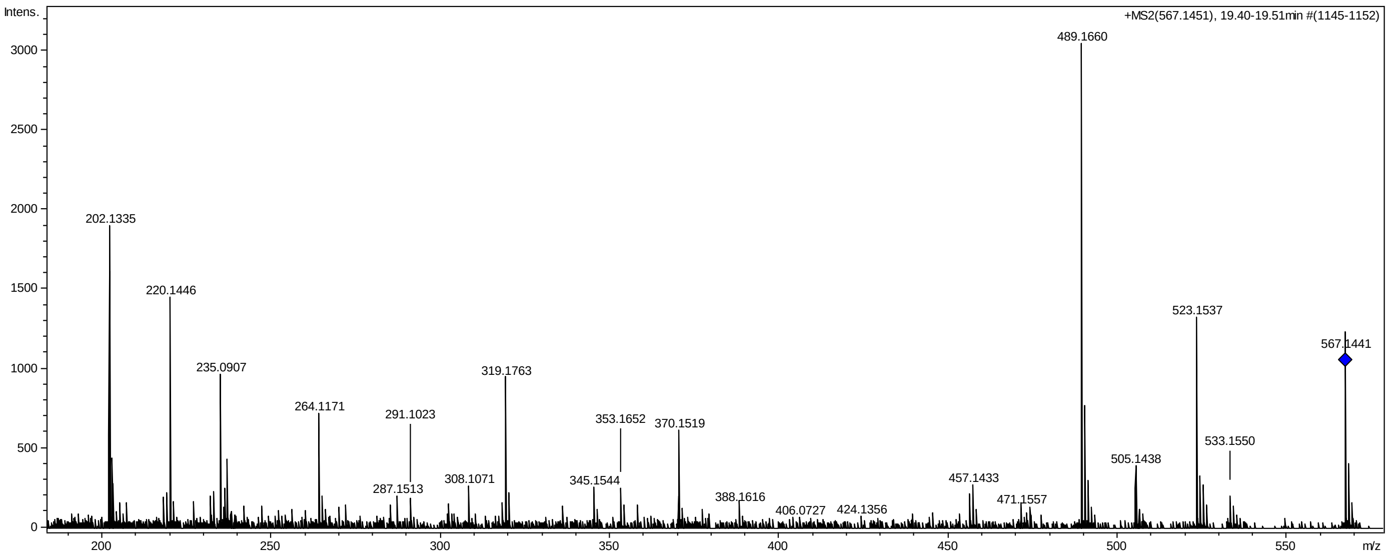

Intens.
+MS2(567.1451), 19.40-19.51min #(1145-1152)
3000
2500
2000
1500
1000
500
0
489.1660
202.1335
220.1446
523.1537
567.1441
235.0907
319.1763
264.1171
291.1023
353.1652
370.1519
533.1550
505.1438
287.1513
308.1071
345.1544
457.1433
388.1616
471.1557
406.0727
424.1356
200
250
300
350
400
450
500
550
m/z

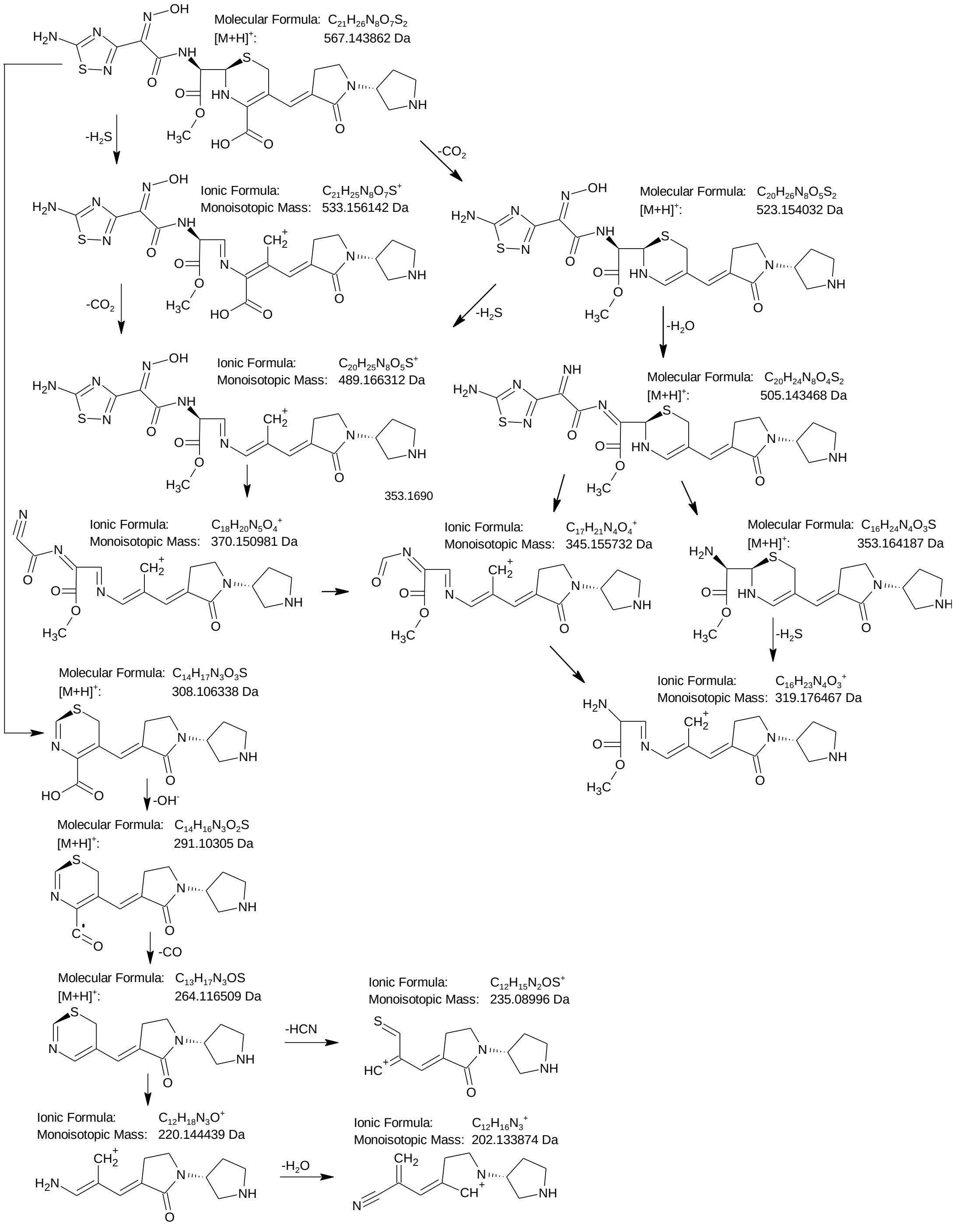

Molecular Formula: $C_{21}H_{26}N_8O_7S_2$
$[M+H]^+$: 567.143862 Da
$-H_2S$
$-CO_2$
Ionic Formula: $C_{21}H_{25}N_8O_7S^+$
Monoisotopic Mass: 533.156142 Da
Molecular Formula: $C_{20}H_{26}N_8O_5S_2$
$[M+H]^+$: 523.154032 Da
$-CO_2$
$-H_2S$
$-H_2O$
Ionic Formula: $C_{20}H_{25}N_8O_5S^+$
Monoisotopic Mass: 489.166312 Da
Molecular Formula: $C_{20}H_{24}N_8O_4S_2$
$[M+H]^+$: 505.143468 Da
353.1690
Ionic Formula: $C_{18}H_{20}N_5O_4^+$
Monoisotopic Mass: 370.150981 Da
Ionic Formula: $C_{17}H_{21}N_4O_4^+$
Monoisotopic Mass: 345.155732 Da
Molecular Formula: $C_{16}H_{24}N_4O_3S$
$[M+H]^+$: 353.164187 Da
$-H_2S$
Molecular Formula: $C_{14}H_{17}N_3O_3S$
$[M+H]^+$: 308.106338 Da
Ionic Formula: $C_{16}H_{23}N_4O_3^+$
Monoisotopic Mass: 319.176467 Da
$-OH^-$
Molecular Formula: $C_{14}H_{16}N_3O_2S$
$[M+H]^+$: 291.10305 Da
-CO
Molecular Formula: $C_{13}H_{17}N_3OS$
$[M+H]^+$: 264.116509 Da
Ionic Formula: $C_{12}H_{15}N_2OS^+$
Monoisotopic Mass: 235.08996 Da
-HCN
Ionic Formula: $C_{12}H_{18}N_3O^+$
Monoisotopic Mass: 220.144439 Da
Ionic Formula: $C_{12}H_{16}N_3^+$
Monoisotopic Mass: 202.133874 Da
$-H_2O$

C. SBP-2 (*m/z* 591)

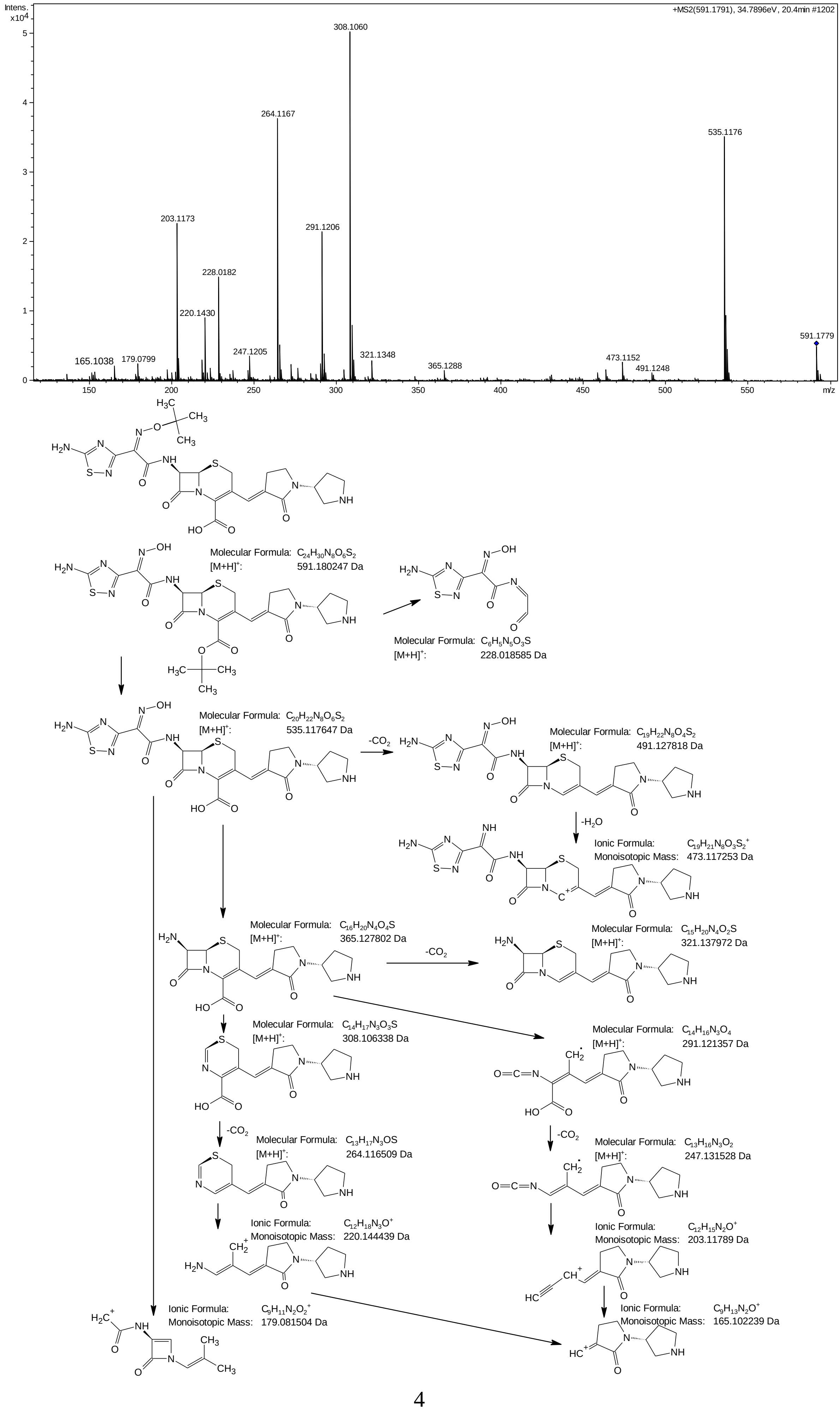

Intens.
x10^4
+MS2(591.1791), 34.7896eV, 20.4min #1202
308.1060
264.1167
535.1176
203.1173
291.1206
228.0182
220.1430
591.1779
247.1205
321.1348
473.1152
165.1038
179.0799
365.1288
491.1248
m/z
Molecular Formula: C24H30N8O6S2
[M+H]+: 591.180247 Da
Molecular Formula: C6H5N5O3S
[M+H]+: 228.018585 Da
Molecular Formula: C20H22N8O6S2
[M+H]+: 535.117647 Da
-CO2
Molecular Formula: C19H22N8O4S2
[M+H]+: 491.127818 Da
-H2O
Ionic Formula: C19H21N8O3S2+
Monoisotopic Mass: 473.117253 Da
Molecular Formula: C16H20N4O4S
[M+H]+: 365.127802 Da
-CO2
Molecular Formula: C15H20N4O2S
[M+H]+: 321.137972 Da
Molecular Formula: C14H17N3O3S
[M+H]+: 308.106338 Da
Molecular Formula: C14H16N3O4
[M+H]+: 291.121357 Da
-CO2
Molecular Formula: C13H17N3OS
[M+H]+: 264.116509 Da
-CO2
Molecular Formula: C13H16N3O2
[M+H]+: 247.131528 Da
Ionic Formula: C12H18N3O+
Monoisotopic Mass: 220.144439 Da
Ionic Formula: C12H15N2O+
Monoisotopic Mass: 203.11789 Da
Ionic Formula: C9H11N2O2+
Monoisotopic Mass: 179.081504 Da
Ionic Formula: C9H13N2O+
Monoisotopic Mass: 165.102239 Da

D. SBP-3 (*m/z* 701)

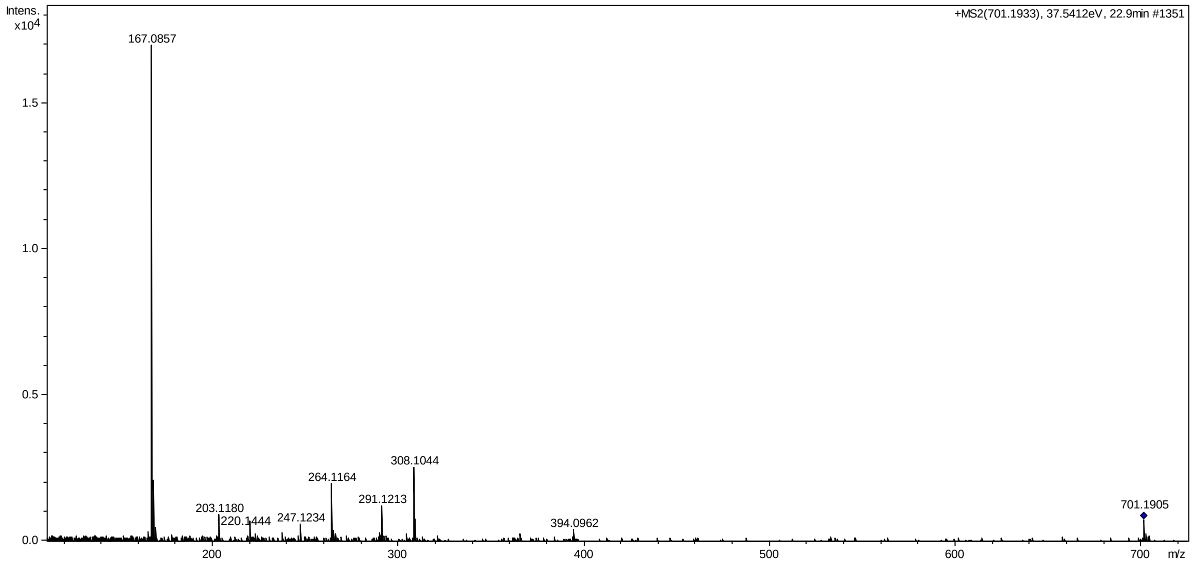

Intens.
x10^4
+MS2(701.1933), 37.5412eV, 22.9min #1351
167.0857
308.1044
264.1164
291.1213
203.1180
220.1444
247.1234
394.0962
701.1905
1.5
1.0
0.5
0.0
200
300
400
500
600
700
m/z

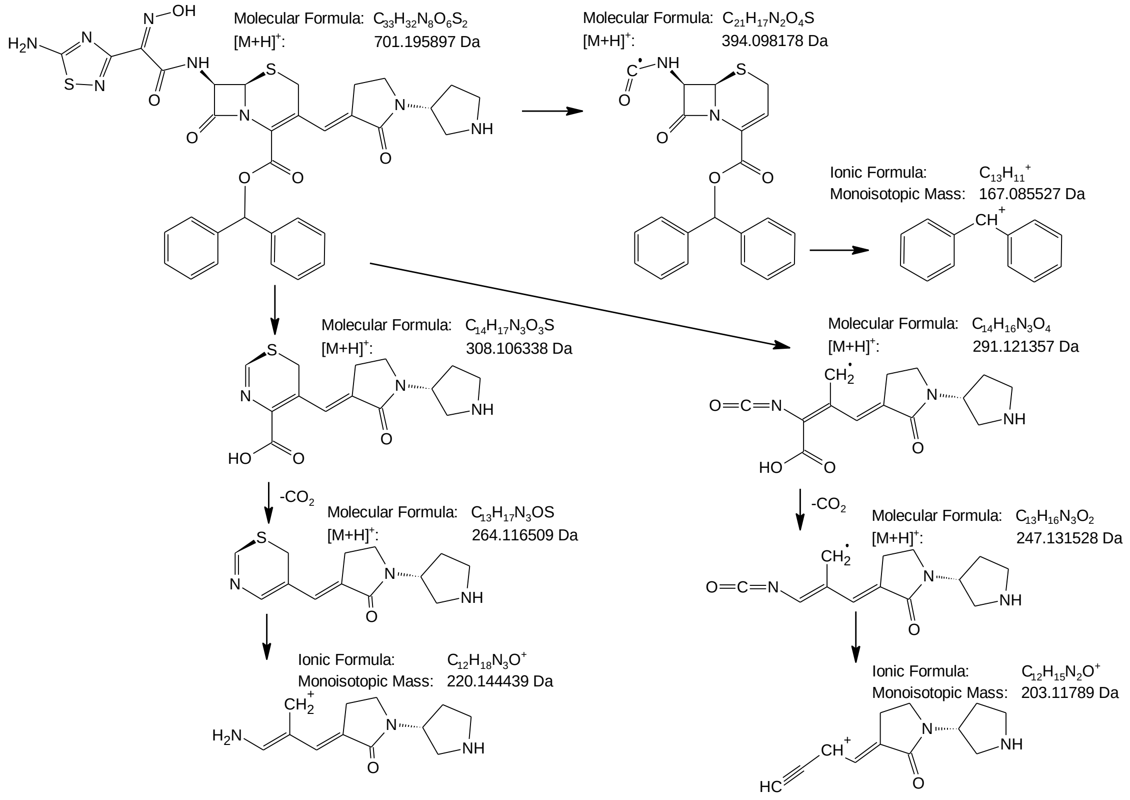

Molecular Formula: C33H32N8O6S2
[M+H]+: 701.195897 Da
Molecular Formula: C21H17N2O4S
[M+H]+: 394.098178 Da
Ionic Formula: C13H11+
Monoisotopic Mass: 167.085527 Da
Molecular Formula: C14H17N3O3S
[M+H]+: 308.106338 Da
Molecular Formula: C14H16N3O4
[M+H]+: 291.121357 Da
-CO2
Molecular Formula: C13H17N3OS
[M+H]+: 264.116509 Da
-CO2
Molecular Formula: C13H16N3O2
[M+H]+: 247.131528 Da
Ionic Formula: C12H18N3O+
Monoisotopic Mass: 220.144439 Da
Ionic Formula: C12H15N2O+
Monoisotopic Mass: 203.11789 Da

E. ADP-0 (*m/z* 538)

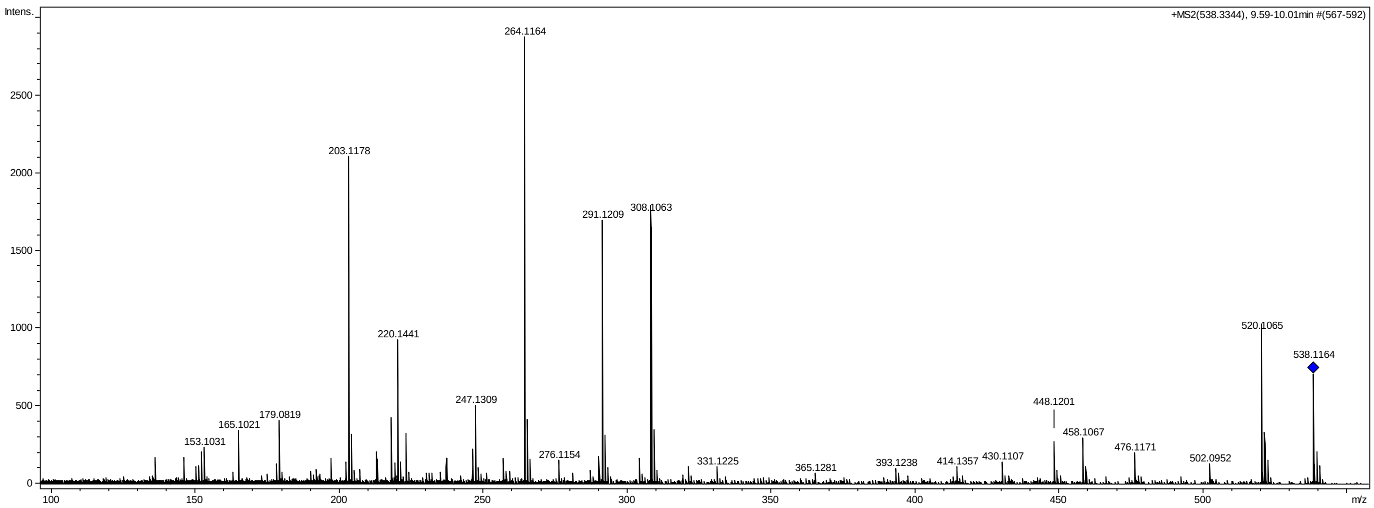

Intens.
+MS2(538.3344), 9.59-10.01min #(567-592)
264.1164
203.1178
291.1209
308.1063
220.1441
520.1065
538.1164
247.1309
448.1201
458.1067
476.1171
179.0819
165.1021
153.1031
276.1154
331.1225
365.1281
393.1238
414.1357
430.1107
502.0952
m/z

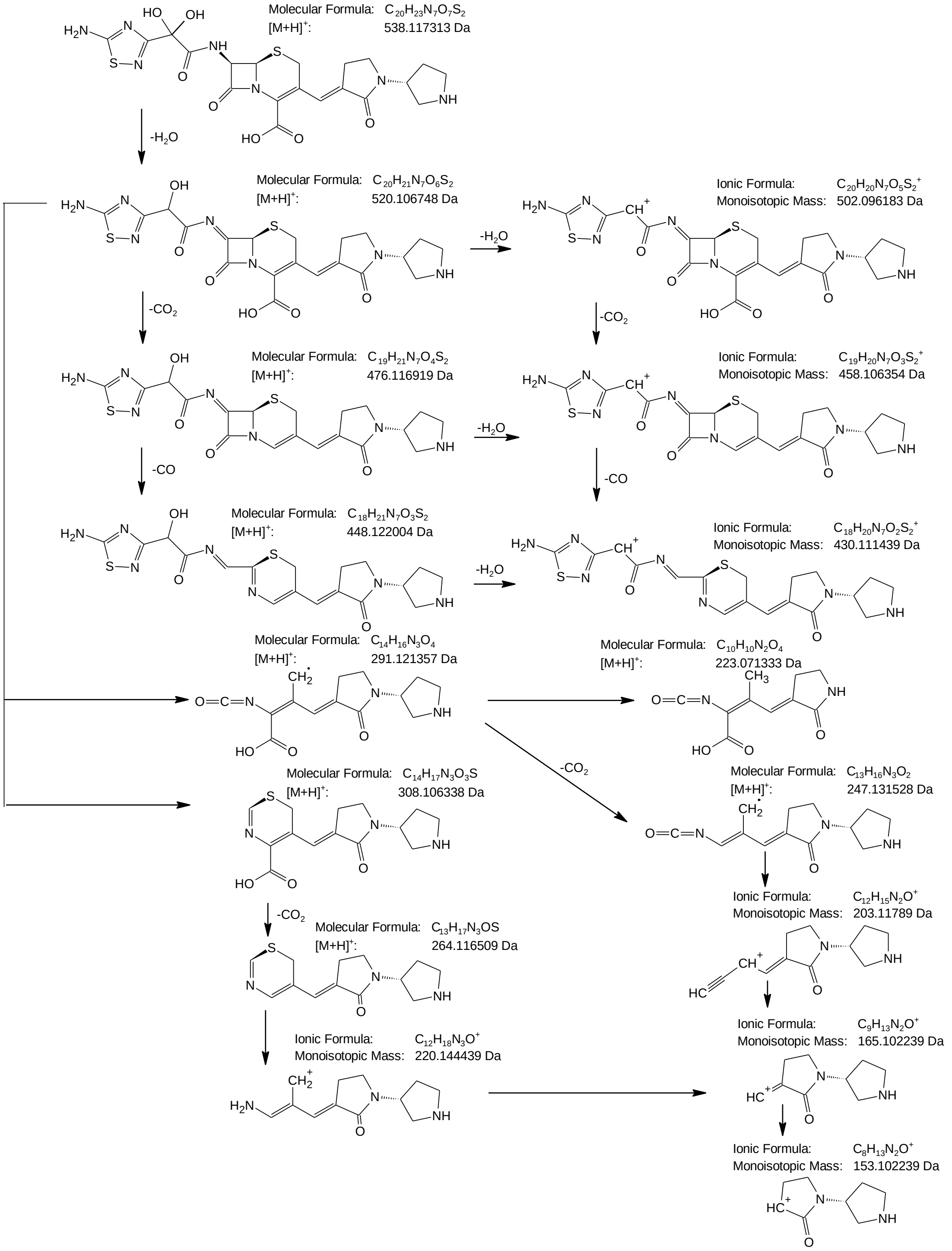

Molecular Formula: C20H23N7O7S2
[M+H]+: 538.117313 Da
-H2O
Molecular Formula: C20H21N7O6S2
[M+H]+: 520.106748 Da
Ionic Formula: C20H20N7O5S2+
Monoisotopic Mass: 502.096183 Da
-CO2
Molecular Formula: C19H21N7O4S2
[M+H]+: 476.116919 Da
Ionic Formula: C19H20N7O3S2+
Monoisotopic Mass: 458.106354 Da
-CO
Molecular Formula: C18H21N7O3S2
[M+H]+: 448.122004 Da
Ionic Formula: C18H20N7O2S2+
Monoisotopic Mass: 430.111439 Da
Molecular Formula: C14H16N3O4
[M+H]+: 291.121357 Da
Molecular Formula: C10H10N2O4
[M+H]+: 223.071333 Da
Molecular Formula: C14H17N3O3S
[M+H]+: 308.106338 Da
Molecular Formula: C13H16N3O2
[M+H]+: 247.131528 Da
Molecular Formula: C13H17N3OS
[M+H]+: 264.116509 Da
Ionic Formula: C12H15N2O+
Monoisotopic Mass: 203.11789 Da
Ionic Formula: C12H18N3O+
Monoisotopic Mass: 220.144439 Da
Ionic Formula: C9H13N2O+
Monoisotopic Mass: 165.102239 Da
Ionic Formula: C8H13N2O+
Monoisotopic Mass: 153.102239 Da

F. ADP-3 (*m/z* 479)

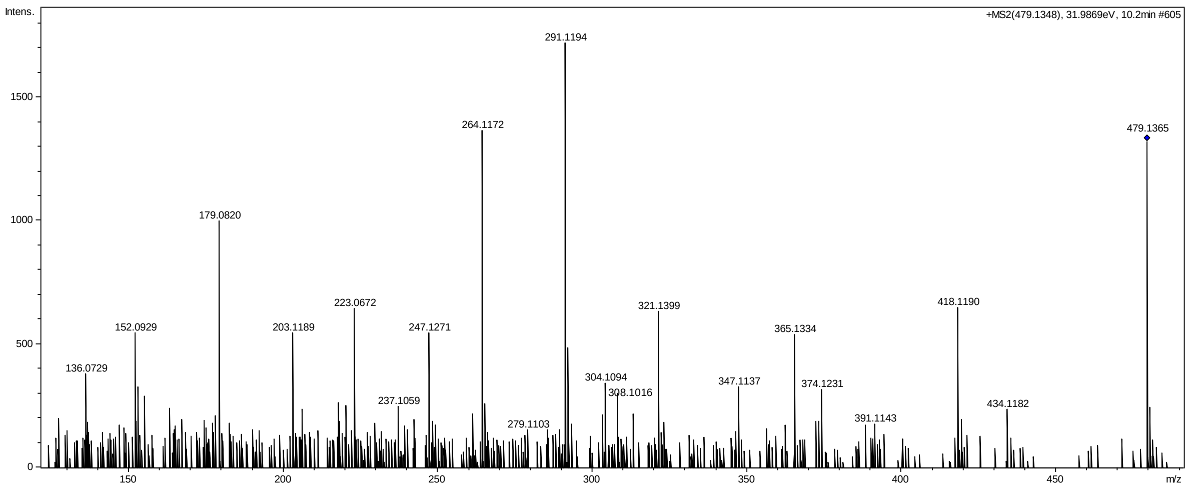
Intens.
+MS2(479.1348), 31.9869eV, 10.2min #605
291.1194
264.1172
479.1365
179.0820
223.0672
321.1399
418.1190
152.0929
203.1189
247.1271
365.1334
136.0729
304.1094
308.1016
347.1137
374.1231
237.1059
279.1103
391.1143
434.1182
m/z

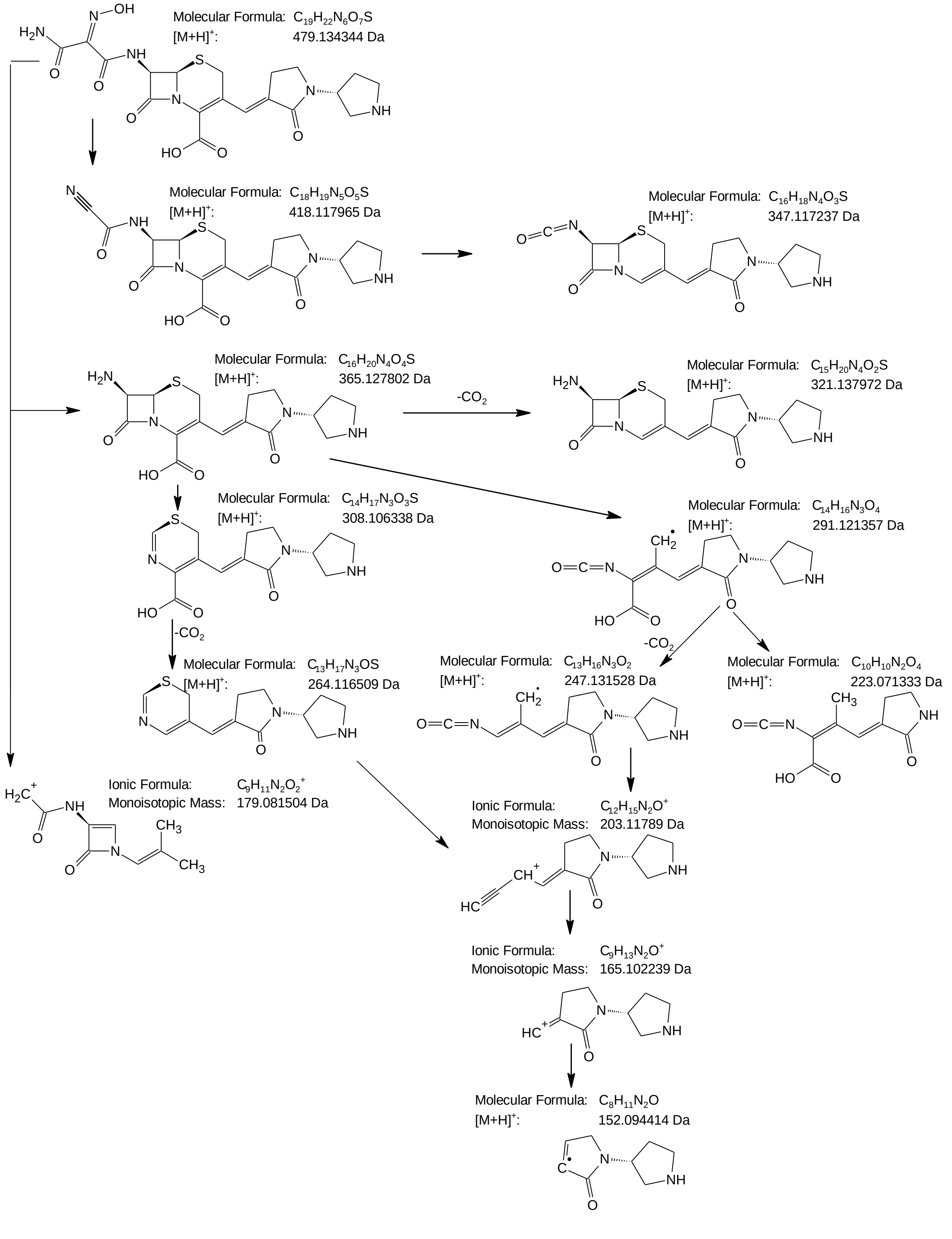
Molecular Formula: $C_{19}H_{22}N_6O_7S$
$[M+H]^+$: 479.134344 Da
Molecular Formula: $C_{18}H_{19}N_5O_5S$
$[M+H]^+$: 418.117965 Da
Molecular Formula: $C_{16}H_{18}N_4O_3S$
$[M+H]^+$: 347.117237 Da
Molecular Formula: $C_{16}H_{20}N_4O_4S$
$[M+H]^+$: 365.127802 Da
$-CO_2$
Molecular Formula: $C_{15}H_{20}N_4O_2S$
$[M+H]^+$: 321.137972 Da
Molecular Formula: $C_{14}H_{17}N_3O_3S$
$[M+H]^+$: 308.106338 Da
Molecular Formula: $C_{14}H_{16}N_3O_4$
$[M+H]^+$: 291.121357 Da
$-CO_2$
Molecular Formula: $C_{13}H_{17}N_3OS$
$[M+H]^+$: 264.116509 Da
$-CO_2$
Molecular Formula: $C_{13}H_{16}N_3O_2$
$[M+H]^+$: 247.131528 Da
Molecular Formula: $C_{10}H_{10}N_2O_4$
$[M+H]^+$: 223.071333 Da
Ionic Formula: $C_9H_{11}N_2O_2^+$
Monoisotopic Mass: 179.081504 Da
Ionic Formula: $C_{12}H_{15}N_2O^+$
Monoisotopic Mass: 203.11789 Da
Ionic Formula: $C_9H_{13}N_2O^+$
Monoisotopic Mass: 165.102239 Da
Molecular Formula: $C_8H_{11}N_2O$
$[M+H]^+$: 152.094414 Da

G. ADP-4 (*m/z* 365)

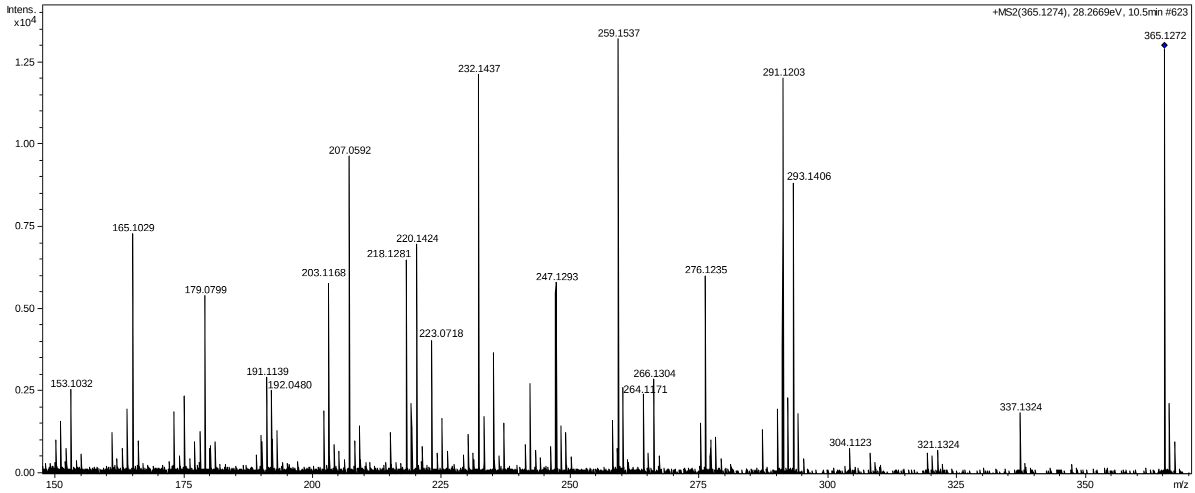

Intens. x10^4
+MS2(365.1274), 28.2669eV, 10.5min #623
1.25
1.00
0.75
0.50
0.25
0.00
365.1272
259.1537
232.1437
291.1203
207.0592
293.1406
165.1029
220.1424
218.1281
203.1168
247.1293
276.1235
179.0799
223.0718
191.1139
192.0480
153.1032
266.1304
264.1171
337.1324
304.1123
321.1324
150
175
200
225
250
275
300
325
350
m/z

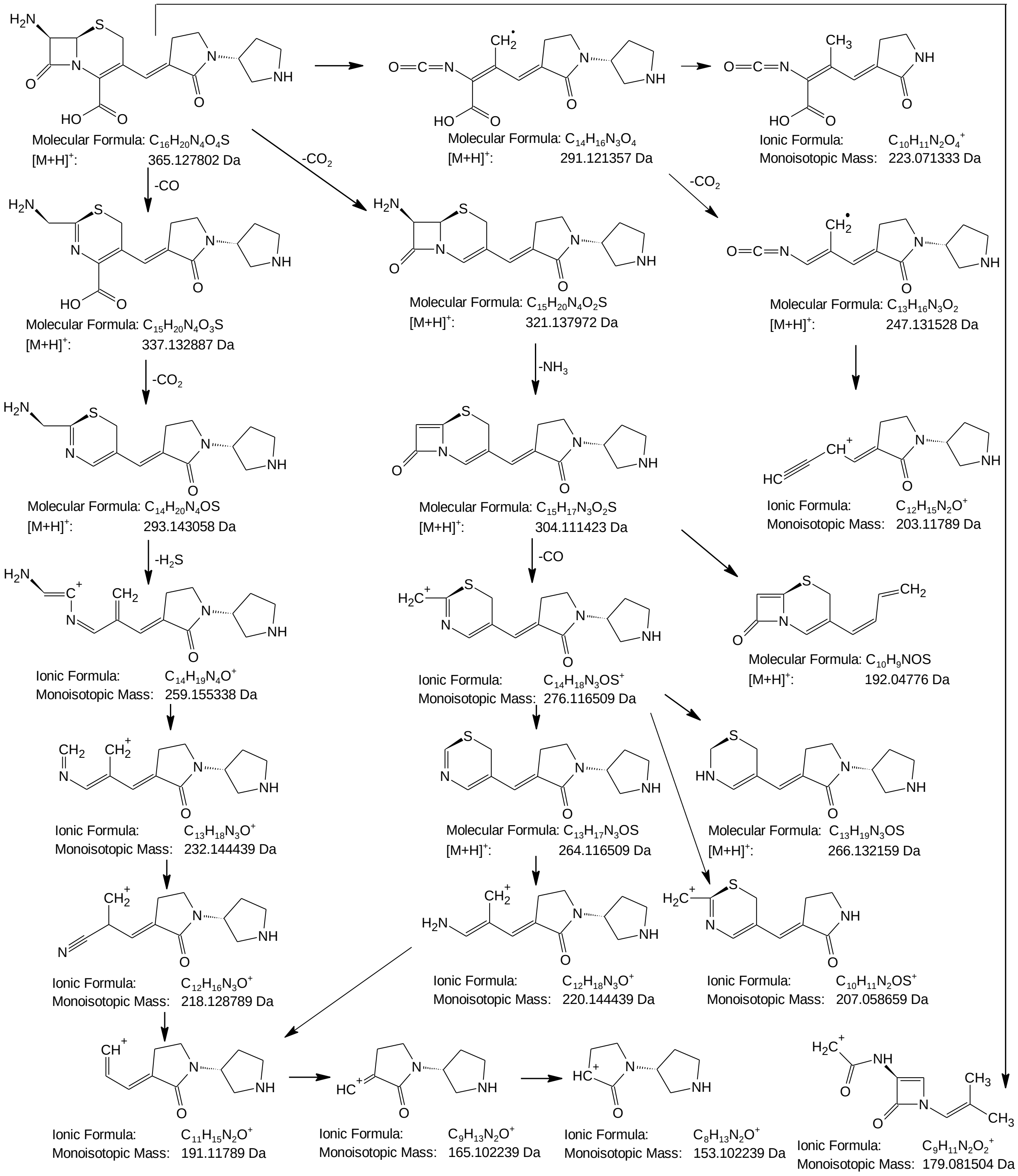

Molecular Formula: $C_{16}H_{20}N_4O_4S$
$[M+H]^+$: 365.127802 Da
-CO
-CO_2
Molecular Formula: $C_{14}H_{16}N_3O_4$
$[M+H]^+$: 291.121357 Da
Ionic Formula: $C_{10}H_{11}N_2O_4^+$
Monoisotopic Mass: 223.071333 Da
-CO_2
Molecular Formula: $C_{15}H_{20}N_4O_3S$
$[M+H]^+$: 337.132887 Da
Molecular Formula: $C_{15}H_{20}N_4O_2S$
$[M+H]^+$: 321.137972 Da
Molecular Formula: $C_{13}H_{16}N_3O_2$
$[M+H]^+$: 247.131528 Da
-CO_2
-NH_3
Molecular Formula: $C_{14}H_{20}N_4OS$
$[M+H]^+$: 293.143058 Da
Molecular Formula: $C_{15}H_{17}N_3O_2S$
$[M+H]^+$: 304.111423 Da
Ionic Formula: $C_{12}H_{15}N_2O^+$
Monoisotopic Mass: 203.11789 Da
-H_2S
-CO
Molecular Formula: $C_{10}H_9NOS$
$[M+H]^+$: 192.04776 Da
Ionic Formula: $C_{14}H_{19}N_4O^+$
Monoisotopic Mass: 259.155338 Da
Ionic Formula: $C_{14}H_{18}N_3OS^+$
Monoisotopic Mass: 276.116509 Da
Ionic Formula: $C_{13}H_{18}N_3O^+$
Monoisotopic Mass: 232.144439 Da
Molecular Formula: $C_{13}H_{17}N_3OS$
$[M+H]^+$: 264.116509 Da
Molecular Formula: $C_{13}H_{19}N_3OS$
$[M+H]^+$: 266.132159 Da
Ionic Formula: $C_{12}H_{16}N_3O^+$
Monoisotopic Mass: 218.128789 Da
Ionic Formula: $C_{12}H_{18}N_3O^+$
Monoisotopic Mass: 220.144439 Da
Ionic Formula: $C_{10}H_{11}N_2OS^+$
Monoisotopic Mass: 207.058659 Da
Ionic Formula: $C_{11}H_{15}N_2O^+$
Monoisotopic Mass: 191.11789 Da
Ionic Formula: $C_9H_{13}N_2O^+$
Monoisotopic Mass: 165.102239 Da
Ionic Formula: $C_8H_{13}N_2O^+$
Monoisotopic Mass: 153.102239 Da
Ionic Formula: $C_9H_{11}N_2O_2^+$
Monoisotopic Mass: 179.081504 Da

H. ADP-5 (*m/z* 265)

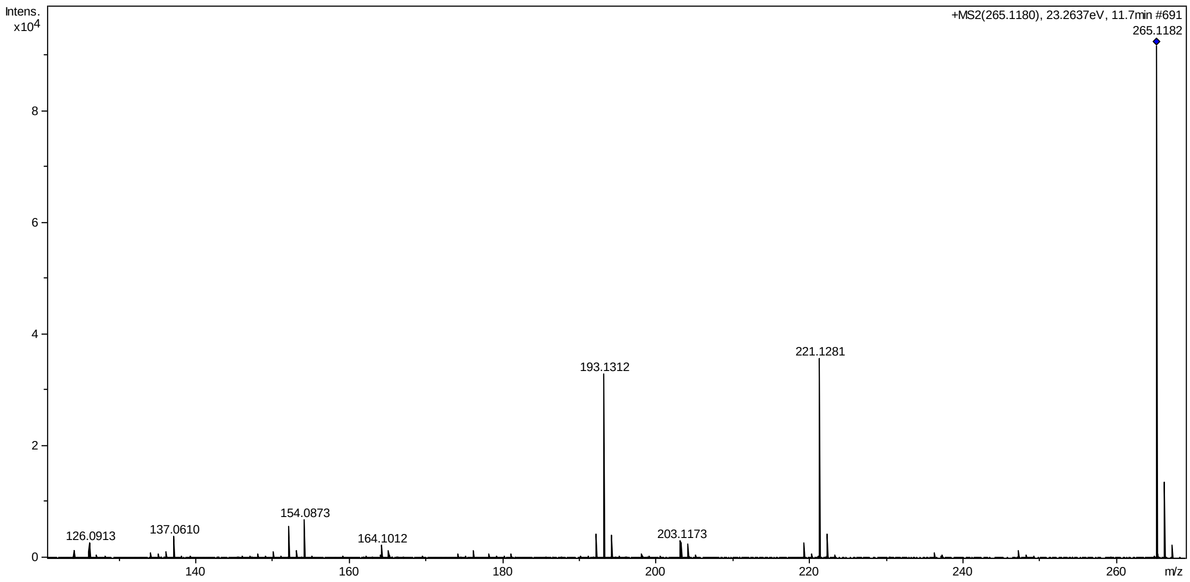

Intens.
x10⁴
+MS2(265.1180), 23.2637eV, 11.7min #691
265.1182
221.1281
193.1312
154.0873
137.0610
126.0913
164.1012
203.1173
m/z

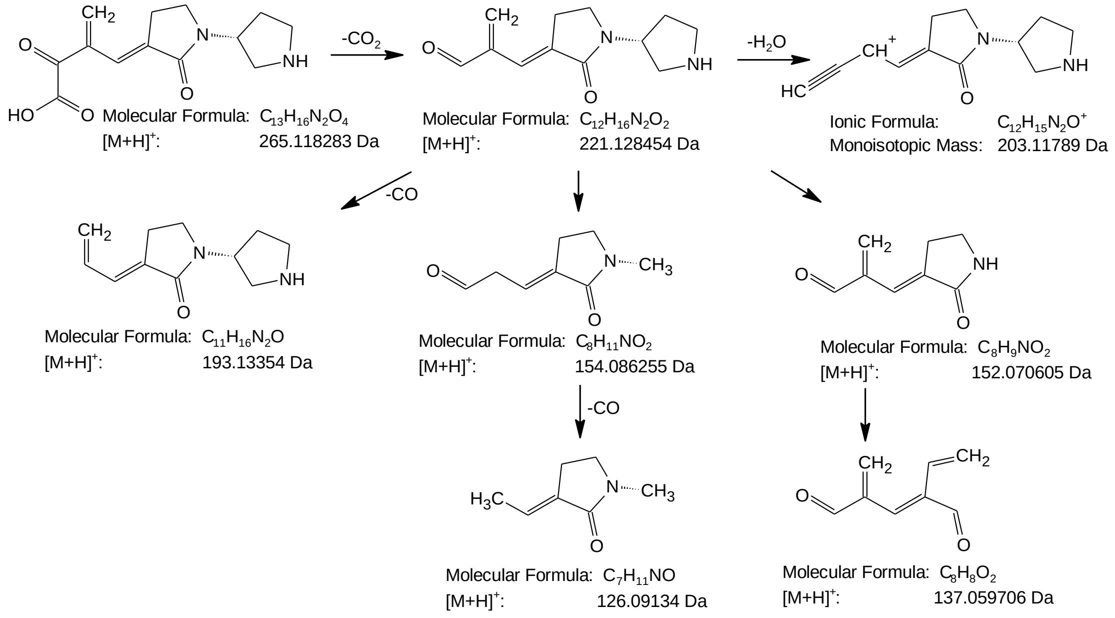

-CO2
-H2O
-CO
-CO
Molecular Formula: C13H16N2O4
[M+H]+: 265.118283 Da
Molecular Formula: C12H16N2O2
[M+H]+: 221.128454 Da
Ionic Formula: C12H15N2O+
Monoisotopic Mass: 203.11789 Da
Molecular Formula: C11H16N2O
[M+H]+: 193.13354 Da
Molecular Formula: C8H11NO2
[M+H]+: 154.086255 Da
Molecular Formula: C8H9NO2
[M+H]+: 152.070605 Da
Molecular Formula: C7H11NO
[M+H]+: 126.09134 Da
Molecular Formula: C8H8O2
[M+H]+: 137.059706 Da

I. ADP-6 (*m/z* 281)

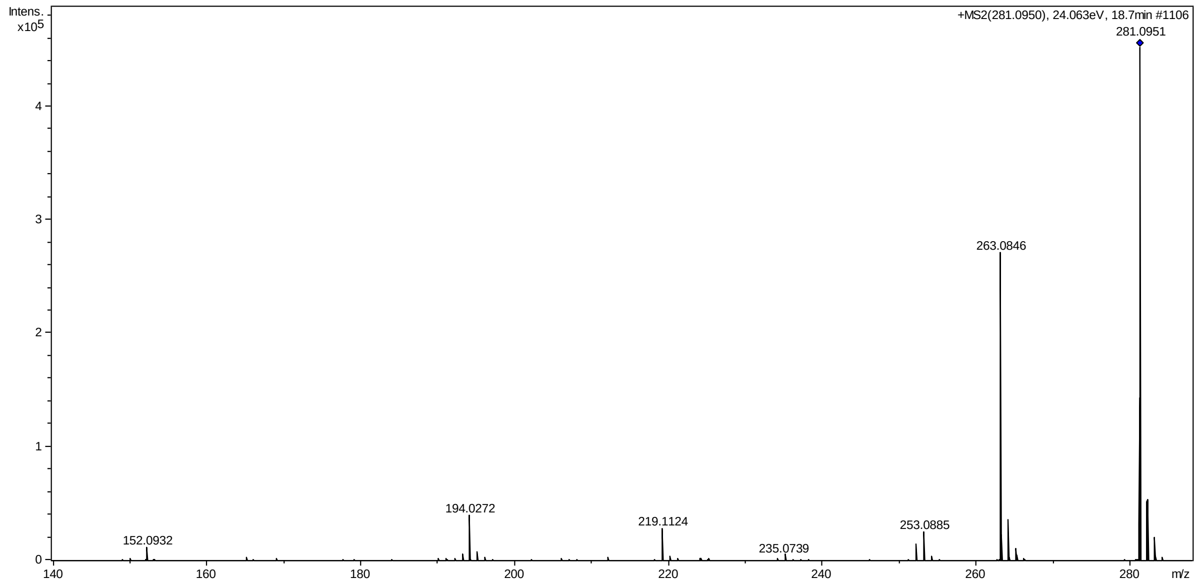

Intens.
x10^5
+MS2(281.0950), 24.063eV, 18.7min #1106
281.0951
263.0846
194.0272
219.1124
253.0885
152.0932
235.0739
m/z

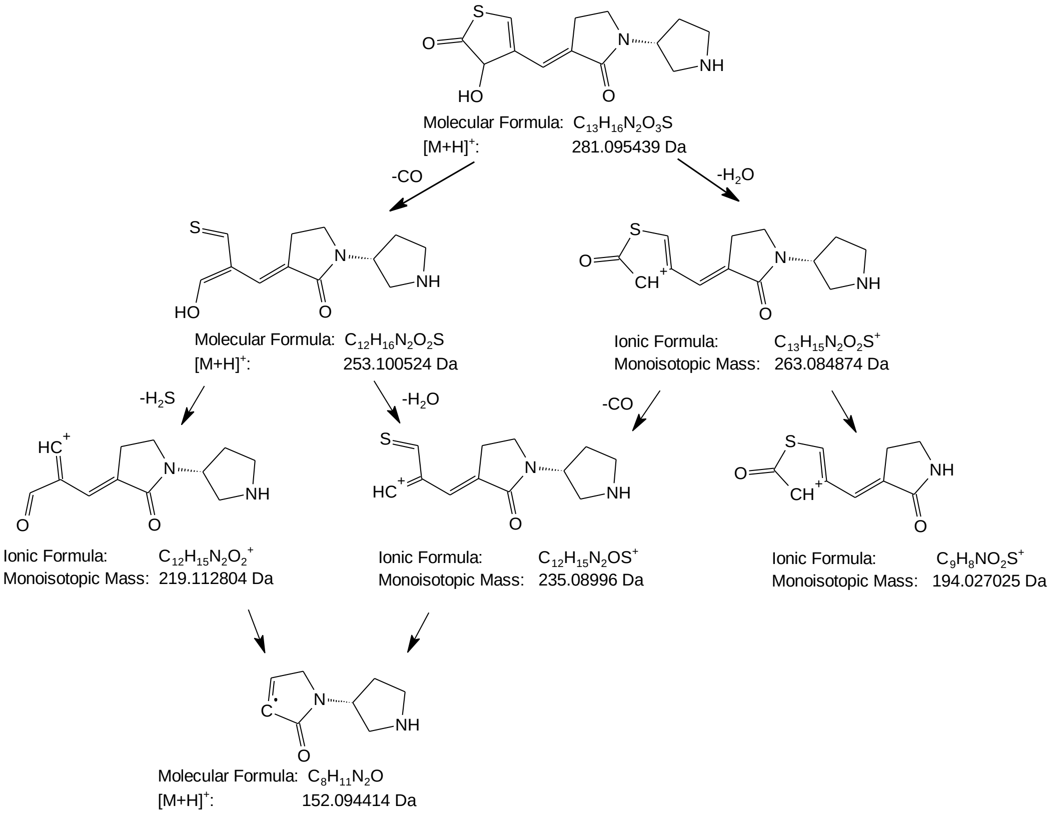

Molecular Formula: $C_{13}H_{16}N_2O_3S$
$[M+H]^+$: 281.095439 Da
-CO
$-H_2O$
Molecular Formula: $C_{12}H_{16}N_2O_2S$
$[M+H]^+$: 253.100524 Da
Ionic Formula: $C_{13}H_{15}N_2O_2S^+$
Monoisotopic Mass: 263.084874 Da
$-H_2S$
$-H_2O$
-CO
Ionic Formula: $C_{12}H_{15}N_2O_2^+$
Monoisotopic Mass: 219.112804 Da
Ionic Formula: $C_{12}H_{15}N_2OS^+$
Monoisotopic Mass: 235.08996 Da
Ionic Formula: $C_9H_8NO_2S^+$
Monoisotopic Mass: 194.027025 Da
Molecular Formula: $C_8H_{11}N_2O$
$[M+H]^+$: 152.094414 Da

J. BDP-1 (*m/z* 553)

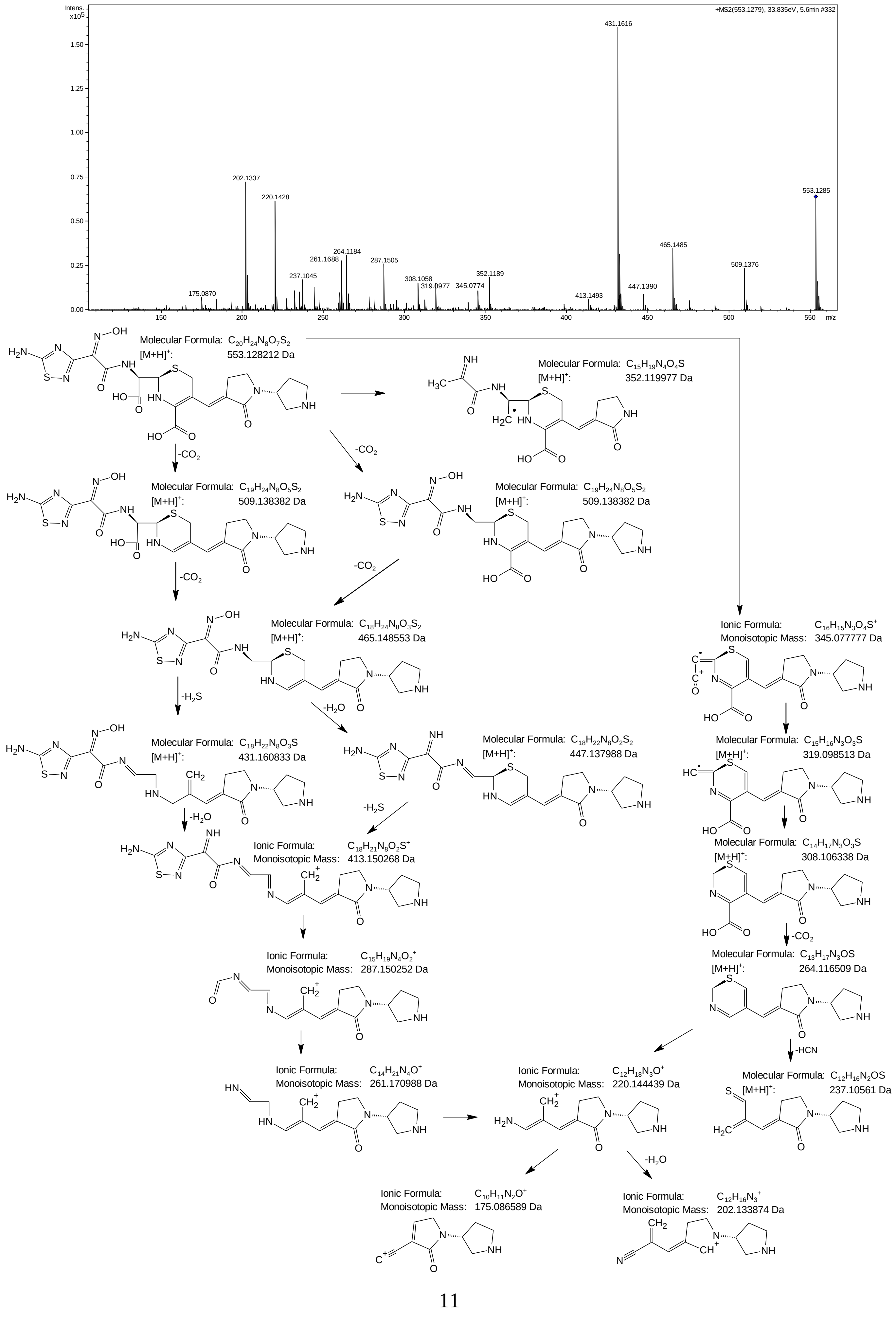
+MS2(553.1279), 33.835eV, 5.6min #332
431.1616
202.1337
220.1428
553.1285
465.1485
509.1376
264.1184
261.1688
287.1505
237.1045
352.1189
308.1058
319.0977
345.0774
175.0870
413.1493
447.1390
Molecular Formula: C20H24N8O7S2
[M+H]+: 553.128212 Da
Molecular Formula: C15H19N4O4S
[M+H]+: 352.119977 Da
-CO2
Molecular Formula: C19H24N8O5S2
[M+H]+: 509.138382 Da
Molecular Formula: C18H24N8O3S2
[M+H]+: 465.148553 Da
Ionic Formula: C16H15N3O4S+
Monoisotopic Mass: 345.077777 Da
-H2S
-H2O
Molecular Formula: C18H22N8O3S
[M+H]+: 431.160833 Da
Molecular Formula: C18H22N8O2S2
[M+H]+: 447.137988 Da
Molecular Formula: C15H16N3O3S
[M+H]+: 319.098513 Da
Ionic Formula: C18H21N8O2S+
Monoisotopic Mass: 413.150268 Da
Molecular Formula: C14H17N3O3S
[M+H]+: 308.106338 Da
Ionic Formula: C15H19N4O2+
Monoisotopic Mass: 287.150252 Da
Molecular Formula: C13H17N3OS
[M+H]+: 264.116509 Da
-HCN
Ionic Formula: C14H21N4O+
Monoisotopic Mass: 261.170988 Da
Ionic Formula: C12H18N3O+
Monoisotopic Mass: 220.144439 Da
Molecular Formula: C12H16N2OS
[M+H]+: 237.10561 Da
Ionic Formula: C10H11N2O+
Monoisotopic Mass: 175.086589 Da
Ionic Formula: C12H16N3+
Monoisotopic Mass: 202.133874 Da

K. BDP-2 (*m/z* 535)

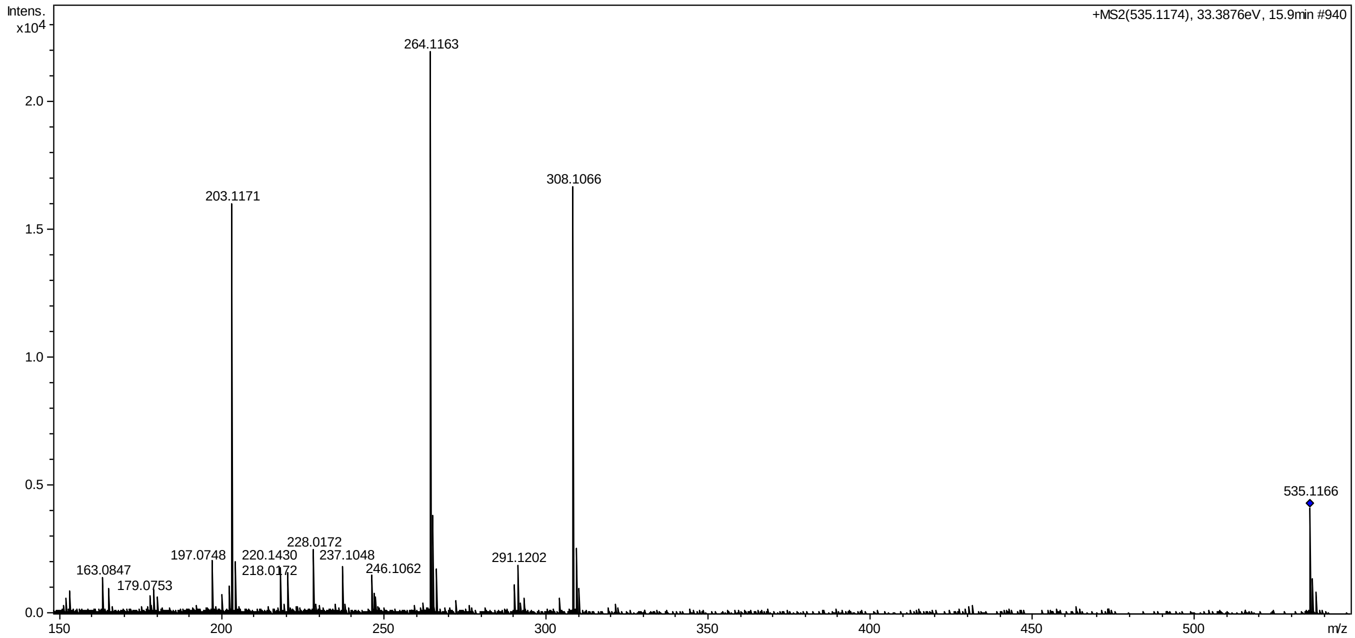

Intens.
x10^4
+MS2(535.1174), 33.3876eV, 15.9min #940
2.0
1.5
1.0
0.5
0.0
264.1163
308.1066
203.1171
535.1166
163.0847
179.0753
197.0748
220.1430
218.0172
228.0172
237.1048
246.1062
291.1202
150
200
250
300
350
400
450
500
m/z

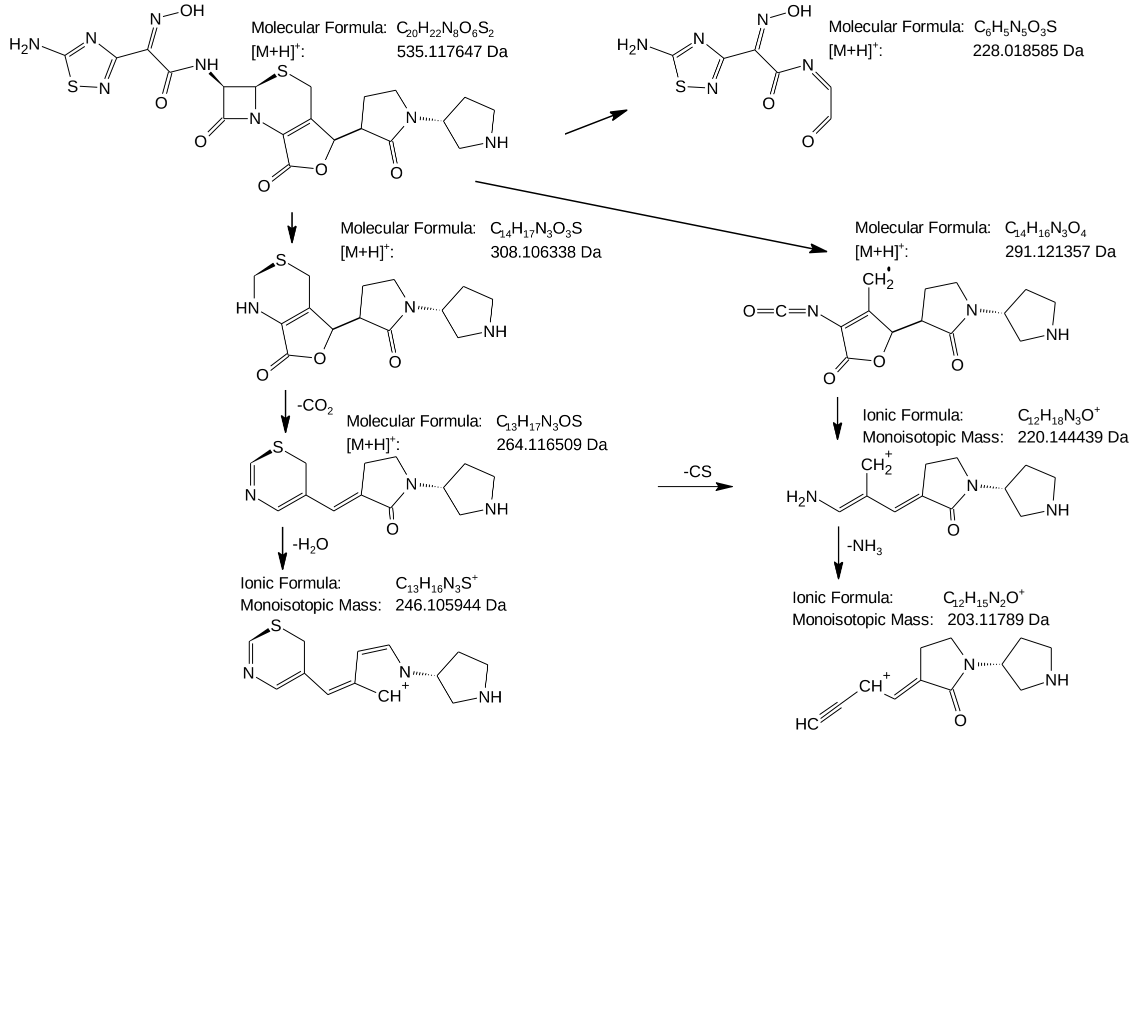

Molecular Formula: $C_{20}H_{22}N_8O_6S_2$
$[M+H]^+$: 535.117647 Da
Molecular Formula: $C_6H_5N_5O_3S$
$[M+H]^+$: 228.018585 Da
Molecular Formula: $C_{14}H_{17}N_3O_3S$
$[M+H]^+$: 308.106338 Da
Molecular Formula: $C_{14}H_{16}N_3O_4$
$[M+H]^+$: 291.121357 Da
$-CO_2$
Molecular Formula: $C_{13}H_{17}N_3OS$
$[M+H]^+$: 264.116509 Da
-CS
Ionic Formula: $C_{12}H_{18}N_3O^+$
Monoisotopic Mass: 220.144439 Da
$-H_2O$
$-NH_3$
Ionic Formula: $C_{13}H_{16}N_3S^+$
Monoisotopic Mass: 246.105944 Da
Ionic Formula: $C_{12}H_{15}N_2O^+$
Monoisotopic Mass: 203.11789 Da

L. ODP-1 (*m/z* 551)

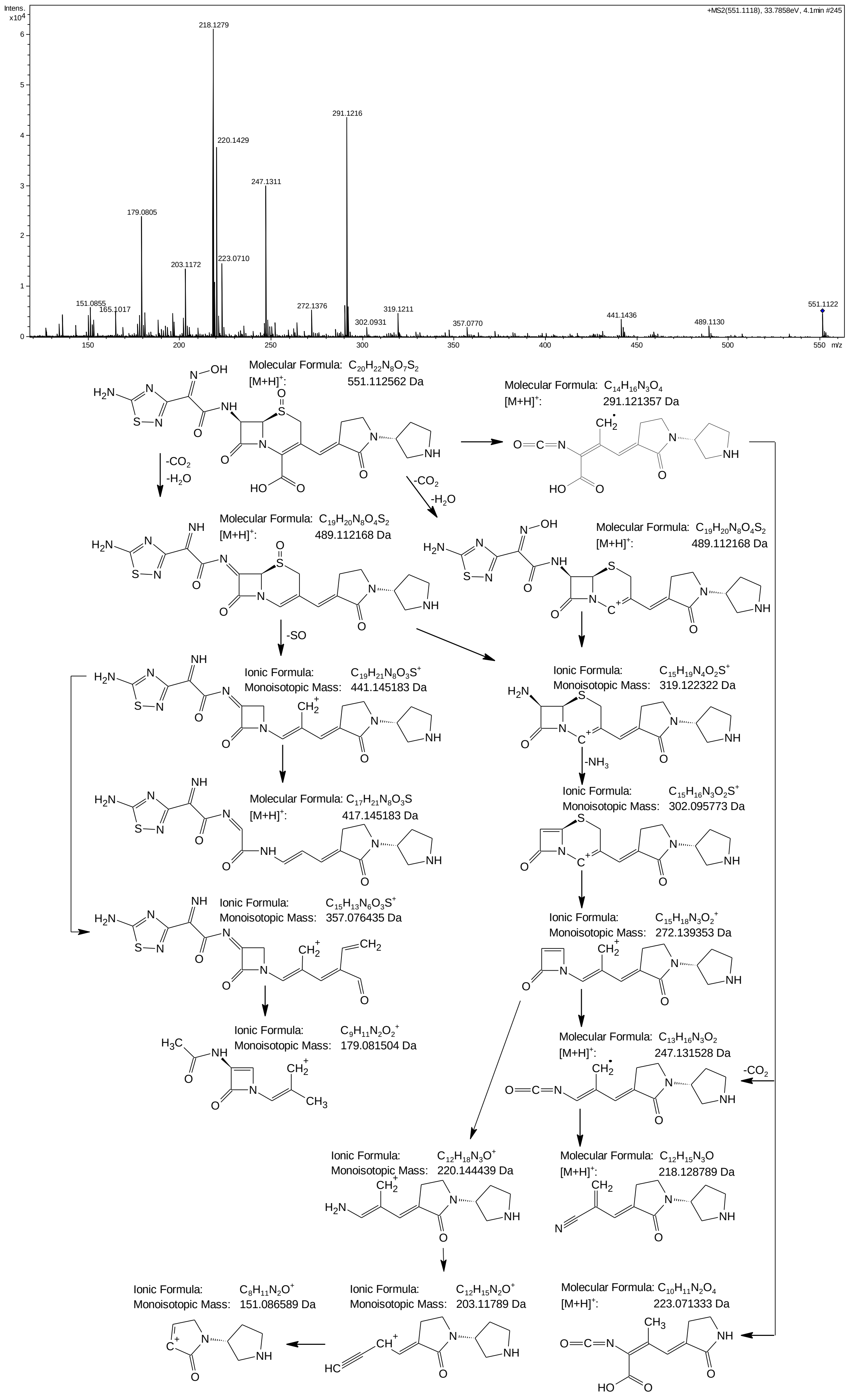
Intens. x10^4
+MS2(551.1118), 33.7858eV, 4.1min #245
218.1279
291.1216
220.1429
247.1311
179.0805
203.1172
223.0710
151.0855
165.1017
272.1376
319.1211
302.0931
357.0770
441.1436
489.1130
551.1122
m/z
Molecular Formula: C20H22N8O7S2
[M+H]+: 551.112562 Da
Molecular Formula: C14H16N3O4
[M+H]+: 291.121357 Da
-CO2
-H2O
Molecular Formula: C19H20N8O4S2
[M+H]+: 489.112168 Da
-SO
Ionic Formula: C19H21N8O3S+
Monoisotopic Mass: 441.145183 Da
Ionic Formula: C15H19N4O2S+
Monoisotopic Mass: 319.122322 Da
-NH3
Molecular Formula: C17H21N8O3S
[M+H]+: 417.145183 Da
Ionic Formula: C15H16N3O2S+
Monoisotopic Mass: 302.095773 Da
Ionic Formula: C15H13N6O3S+
Monoisotopic Mass: 357.076435 Da
Ionic Formula: C15H18N3O2+
Monoisotopic Mass: 272.139353 Da
Ionic Formula: C9H11N2O2+
Monoisotopic Mass: 179.081504 Da
Molecular Formula: C13H16N3O2
[M+H]+: 247.131528 Da
Ionic Formula: C12H18N3O+
Monoisotopic Mass: 220.144439 Da
Molecular Formula: C12H15N3O
[M+H]+: 218.128789 Da
Ionic Formula: C8H11N2O+
Monoisotopic Mass: 151.086589 Da
Ionic Formula: C12H15N2O+
Monoisotopic Mass: 203.11789 Da
Molecular Formula: C10H11N2O4
[M+H]+: 223.071333 Da

M. ODP-2 (*m/z* 551)

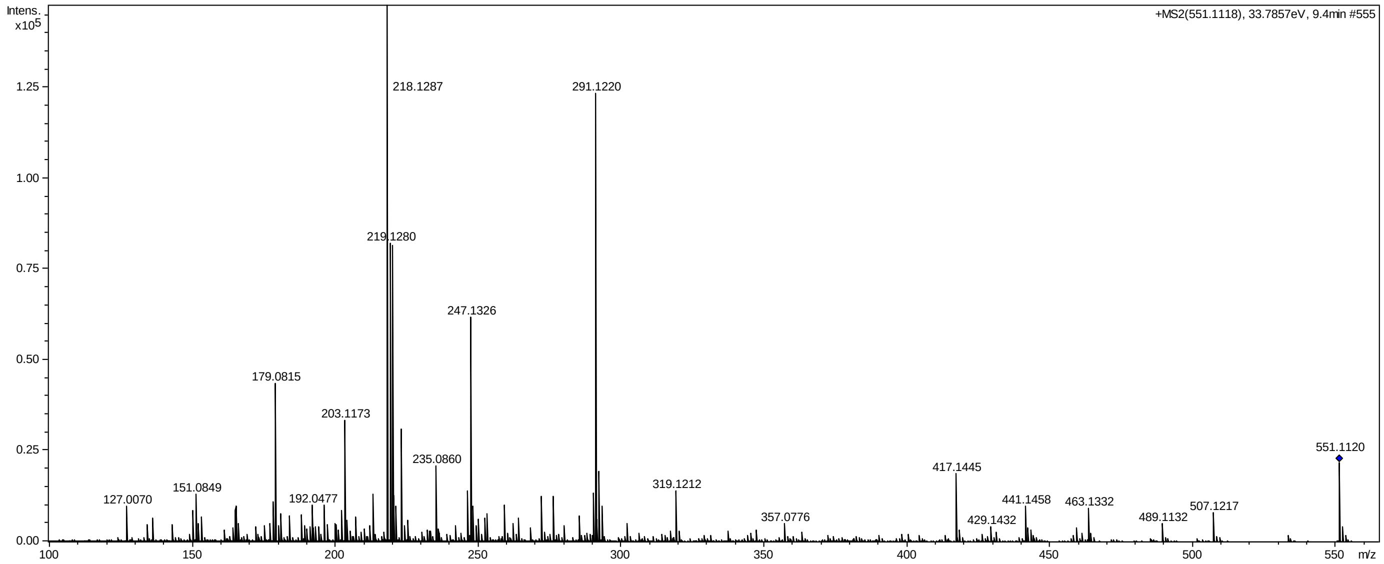

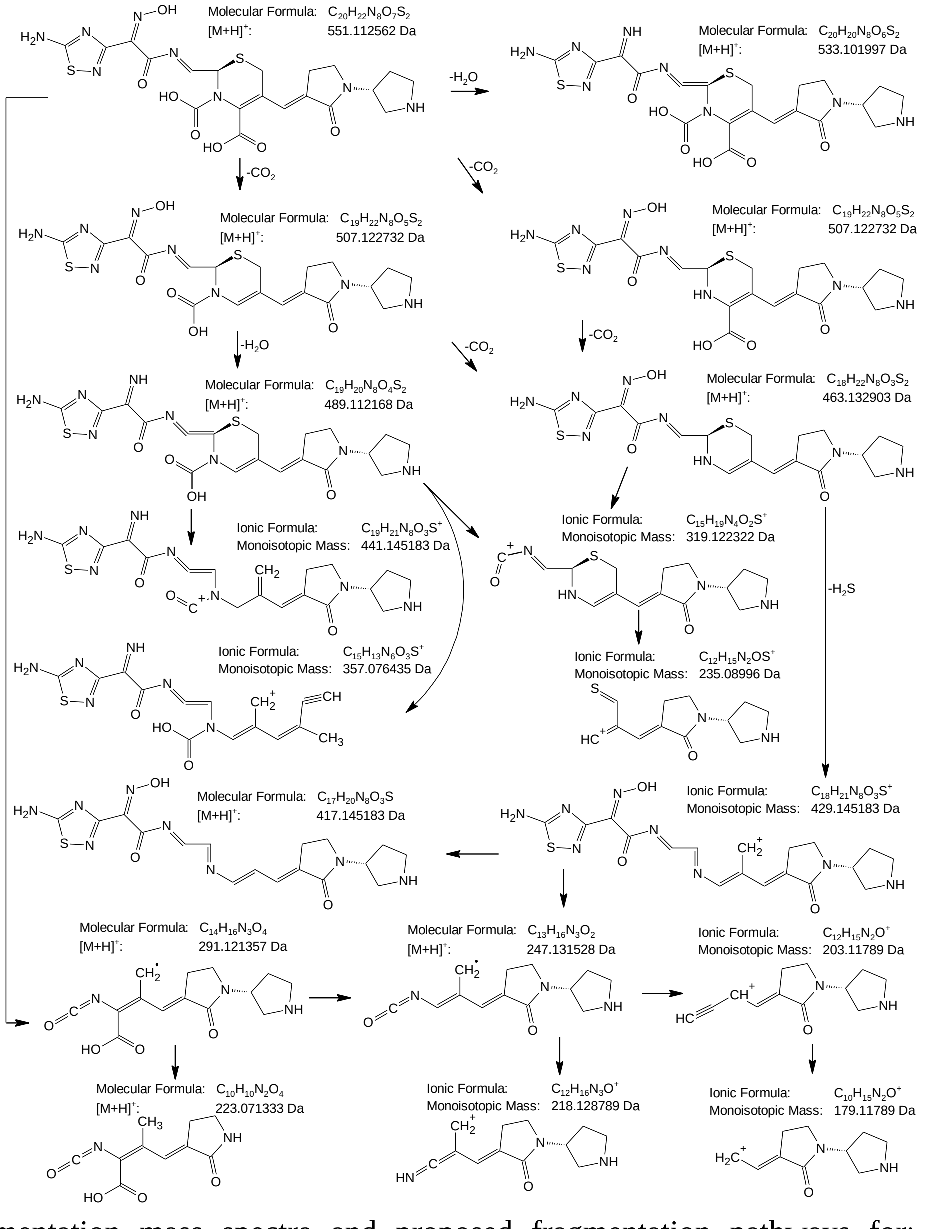

Figure S1. Fragmentation mass spectra and proposed fragmentation pathways for: (A) ceftobiprole, (B) SBP-1, (C) SBP-2 (common for both isomers), (D) SBP-3 (common for both isomers), (E) ADP-0, (F) ADP-3 (common for both isomers), (G) ADP-4, (H) ADP-5, (I) ADP-6, (J) BDP-1 (common for both isomers), (K) BDP-2, (L) ODP-1, (M) ODP-2.

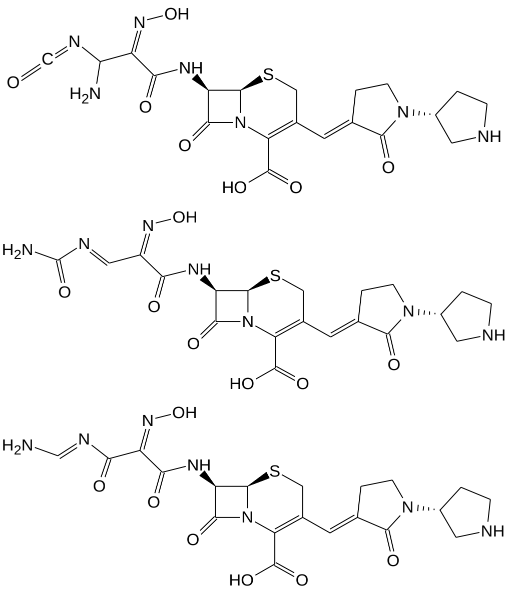

Figure S2. Proposals of the structure of ADP-1.

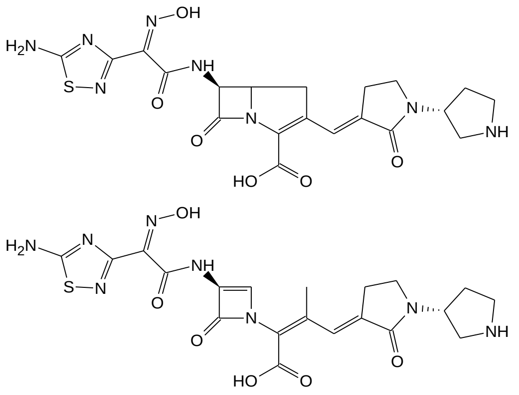

Figure S3. Proposals of the structure of PDP-2.
